# Thermodynamic model of the oxidation of *Ln*-doped UO_2_

**DOI:** 10.1038/s41598-023-42616-x

**Published:** 2023-10-20

**Authors:** V. L. Vinograd, A. A. Bukaemskiy, G. Deissmann, G. Modolo

**Affiliations:** Institute of Energy and Climate Research—Nuclear Waste Management (IEK-6), Forschungszentrum Jülich GmbH, Jülich, Germany

**Keywords:** Geochemistry, Computational methods

## Abstract

*Ln*-doped UO_2_ is often considered as a model system of spent nuclear fuel (SNF) helping to reveal effects of fission and activation products on its chemical stability. Comparing thermodynamics of UO_2_-UO_3_ and *Ln*O_1.5_-UO_2_-UO_3_ systems provides a means to understand the phenomenon of an increased resistivity of *Ln*-doped UO_2_ to oxidation in air relative to pure UO_2_. Here a thermodynamic model is developed and is applied to investigate detailed phase changes occurring along the oxidation of *Ln*-doped fluorite to U_3_O_8_. The study proposes that an enhanced resistivity to oxidation of *Ln*-doped UO_2_ is likely caused by a thermodynamically driven partitioning of *Ln* between a fluorite-type phase and a U_3_O_8_ polymorph, which at ambient temperatures becomes hindered by slow diffusion.

## Introduction

Rare earth elements (REE) such as Nd, Ce, La and Y, occur as high yield fission products in spent nuclear fuel (SNF)^1,2^, while Gd is often added to the fuel as a burnable poison^3^. The occurrence of these elements, colloquially referred here to as “lanthanides” (*Ln*), is very important for understanding thermodynamic differences between SNF and pure UO_2_. These differences may have a profound influence on the chemical stability of SNF in diverse scenarios relevant to its final disposal in a deep geological repository. Although any repository is designed to prevent a contact of spent fuel with ground waters^4^, a possibility of such an interaction is expected after a long time span of ~ 10,000 years. This consideration motivates research on SNF dissolution. Under reducing conditions, pure UO_2_ fluorite has a very low solubility in aqueous solutions^5^. The solubility of UO_2_ is enhanced, however, in the presence of radiolytic oxidants, such as H_2_O_2_, which could be generated at the solid/water interface by alpha-radiation. The high chemical reactivity of H_2_O_2_ then creates locally oxidizing conditions, at which much more soluble oxidized forms of UO_2_ could be formed^6^. Many recent studies of oxidative dissolution of pure UO_2_ and doped UO_2_ solid solutions, including simulated fuels^7–9^, were concerned with the question of whether the fission products and transuranium elements that are contained in SNF enhance or suppress its oxidative dissolution rate in aqueous media. These studies clearly showed that doped UO_2_ dissolves in aqueous solutions at a much lower rate compared to pure UO_2_. Consistently, experiments on electrochemical dissolution of *Ln*-doped UO_2_ and of simulated fuels^10–13^ showed that impurities make UO_2_ significantly less susceptible to oxidation. Also, considerable experimental evidence was accumulated showing that *Ln*-doping and burn-up significantly hinders the kinetics of oxidation of UO_2_ and of UO_2_-based SNF in air^14–18^. Detailed analysis of this data^19^ suggested that air-oxidation and oxidative dissolution studies performed with pure UO_2_ would provide conservative kinetic constraints for the reactivity of a spent UO_2_-based nuclear fuel, while fuel dissolution rates determined with average burnup fuel would be also valid for high burnup fuel. Clearly, these important conclusions would have an even higher value for fuel performance assessments if the mechanism of the enhanced resistivity of doped UO_2_ was fully understood.

However, this mechanism is still discussed controversially. Razdan & Shoesmith^11^ linked the stabilizing effect of *Ln*-doping to the formation of *Ln*-V clusters (V denotes oxygen vacancies), which were thought to reduce the number of vacant sites that could host oxygen anions. The formation of *Ln*-V clusters in reduced *Ln*-doped solid solutions (*Ln* = Y, Dy, Gd, Eu, Sm) was confirmed in a computational study^20^. However, the active role of vacancies in the retardation effect is doubtful as the vacancies must be filled in before a sample could be oxidized to hyper-stoichiometry. Casella et al.^21^ suggested that *Ln*^3+^ dopants due to their effectively negative charge (relative to U^+4^) repel oxygen interstitials thus limiting the fraction of interstitial sites available for accommodating an excess of O^−2^. The electrostatic origin of the retardation is doubtful, however, as the effect is also observed for Th^+4^ ^22^. Kim et al.^16^ proposed that the decreased rates of oxidation in air measured on Gd-doped samples were due to a decreased fraction of U^+4^ caused by the reaction 2U^+4^ = Gd^+3^ + U^+5^. The decrease in the fraction of oxidizable U^+4^ was thought to hinder the incorporation of interstitials. The common difficulty of the discussed hypotheses^11,16,21^ is that the dopants are assumed to alter the lattice of fluorite locally hindering its ability to incorporate oxygen. This assumption implies the oxidative resistivity to be about linearly proportional to the dopant concentration. On the contrary, oxidative dissolution yields drop by orders of a magnitude upon an addition of a few mole percent of a dopant^8,9^.

Solution calorimetry studies^23,24^ provided evidence for a remarkable thermodynamic stability of *Ln*-doped UO_2_ solid solutions with an oxygen to metal (*O/M*) ratio of ~ 2. The formation enthalpy of U_1-z_*Ln*_z_O_2_ compounds (*Ln* = La, Nd, Y) becomes strongly more negative with the *Ln* fraction, *z*, for 0 < *z* < 0.5. The stability of these compounds correlates with an independent observation that *Ln*-doped solid solutions of intermediate compositions (0.5 < *z* < 0.67) synthesized in strongly oxidizing conditions at temperatures of about 1273 K after achieving the stoichiometric relationship of O/M ~ 2 do not oxidize further to hyper-stoichiometry^25–30^. Based on the measured endothermic energies Mazeina et al.^23^ suggested that the activity of the UO_2_ endmember is greatly reduced in doped fluorite solid solutions making them less prone to oxidation. However, this activity estimation assumed the applicability of a regular mixing model to UO_2_–*Ln*O_1.5_ solid solutions. This assumption needs further testing.

A further understanding of the resistivity mechanism could possibly be found within a model that considers not only the fluorite phase, but also other phases that form because of its oxidation. Kinetic factors associated with UO_2_ → U_4_O_9_ and U_4_O_9_ → U_3_O_8_ transformations could be then considered. Particularly, a coupling between thermodynamic and kinetic factors can be investigated. Therefore, here we compare the thermodynamics of oxidation in pure UO_2_ and in *Ln*-doped systems, analyse changes in phase relations induced by the doping and discuss kinetic factors that would likely be enhanced in the doped case. The model is then applied to air oxidation experiments. Oxidative dissolution experiments are also discussed; however, no attempt is made to simulate processes in the aqueous phase.

Consequently, the aim here is to model phase changes occurring in UO_2_-UO_3_ and *Ln*O_1.5_-UO_2_-UO_3_ systems along with changes in the temperature, *T*, the partial pressure of oxygen, ${P}_{{\mathrm{O}}_{2}},$ and the mole fraction, *z*, of *Ln*O_1.5_. The assessment of 17 *Ln*O_1.5_-UO_2_-UO_3_ systems (*Ln* = La–Lu, Y, Sc) is a too ambitious task. Conversely, considering only a single system, e.g., *Ln* = La, is also not a good option because the thermodynamic data would not be sufficient for developing a comprehensive model. Rather, we aim at a generalized model for a typical “*Ln*” that provides a reasonably good description of phase equilibria in many *Ln*O_1.5_-UO_2_-UO_3_ systems, being possibly deficient in some detail, in specific cases. Thus, we feed the model with data on different *Ln*-systems, assuming that differences in thermodynamic properties of lanthanides that are relevant to oxidation reactions can be ignored at a first glance. The emphasis is put on accurately reproducing the effect of *Ln*-doping on the equilibrium oxygen partial pressure by adjusting *relative* Gibbs free energies of relevant endmembers of solid solution phases. The gas phase is not simulated directly; the dependence on the oxygen partial pressure is formally included into the Gibbs free energies of solids. This simple approach is thought sufficient for the present task of evaluating the general effect of a trivalent dopant. The model derivation is mostly built on data on *Ln* = {La, Nd, Gd}. The data on heavier *Ln* are relatively scarce. We also exclude systems with Eu, Ce and Pr since these elements can occur in different oxidation states, making a simple generalized approach not feasible.

The study includes phases with MO_2+*δ*_, M_4_O_9_, and M_3_O_8_ composition. The rhombohedral phases, M_7_O_12_, M_8_O_15_ and M_8_O_16_, are omitted. Only the $Fm\overline{3 }m$ MO_2+*δ*_ phase, colloquially referred here to as “fluorite”, is treated as a non-stoichiometric compound. Because the model performance is illustrated here mostly with data on La and Nd, only the hexagonal (A) polymorph of M_2_O_3_ is considered. The tetragonal U_3_O_7_ phase is also omitted. Tetragonal phases with O/M ratios of ~ 2.3 and ~ 2.33 appear in oxidation experiments with pure UO_2_ at temperatures below ~ 823 K^31^. In doped systems the tetragonal phases are observed rarely. However, a cubic γ-M_4_O_9_ phase with an O/M ratio of ~ 2.4 appears within the same stability range^14^. The present study does not make an attempt to exactly reproduce these complex phase relations. Instead, the observed tendency for the formation of fluorite-like phases with O/M ~ 2.33 is emulated here with the aid of the general fluorite model. The current fluorite phase model builds upon the previous model developed in^32^. The non-stoichiometry interval for MO_2+*δ*_ is extended to −1/2 < *δ* < 1/3. As in^32^, an effort is made not only to describe the Gibbs free energy of the fluorite phase as a function of *T*, ${P}_{{\mathrm{O}}_{2}}$, and *z*, but also to evaluate its lattice parameter as a function of the same variables. This is achieved by coupling the thermodynamic model with an ion-packing model. A detailed formulation is given in the “Methods” section. The results are presented below as a series of phase diagrams.

## Results

### Non-stoichiometry in the fluorite solid solution

**Figure 1:**
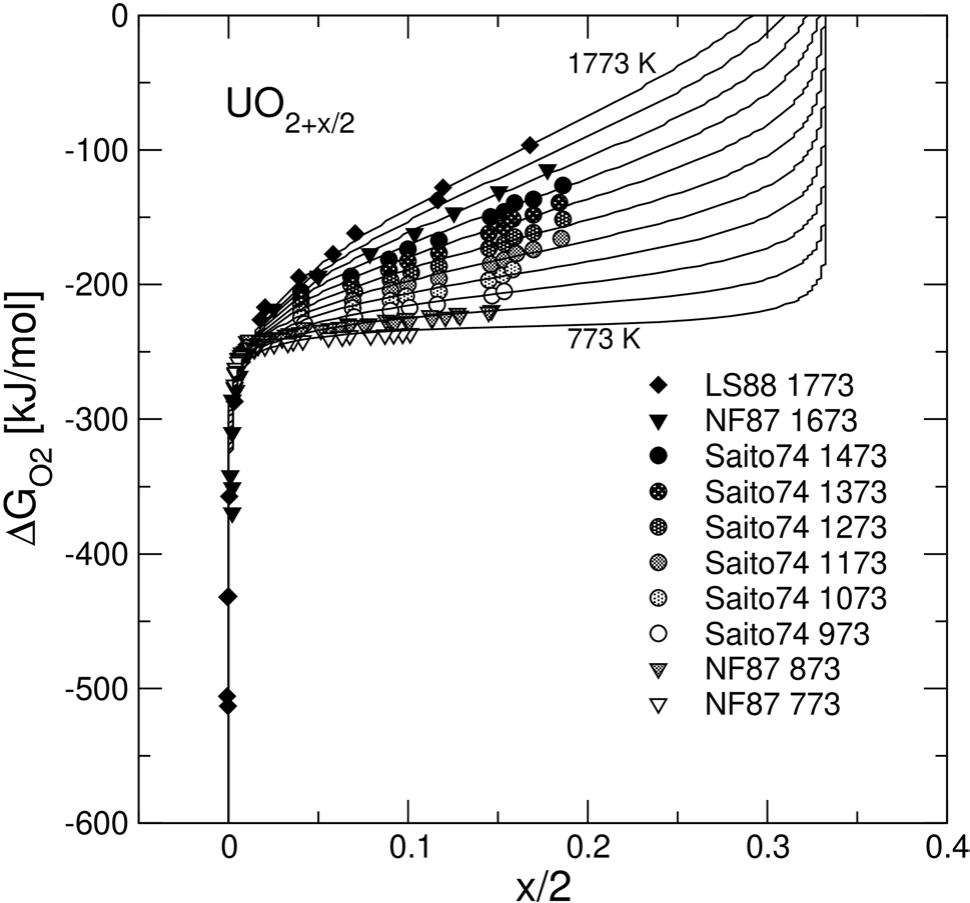
Model fit to the experimental data on ${\Delta G}_{{\mathrm{O}}_{2}}=\mathit{RT}\mathrm{ln}({P}_{{\mathrm{O}}_{2}}/{P}^{0})$ vs. $\delta =x/2$ for pure UO_2_. The experimental data are from Lindemer & Sutton^36^, Nakamura & Fujino^37^ and Saito^38^.

**Figure 2:**
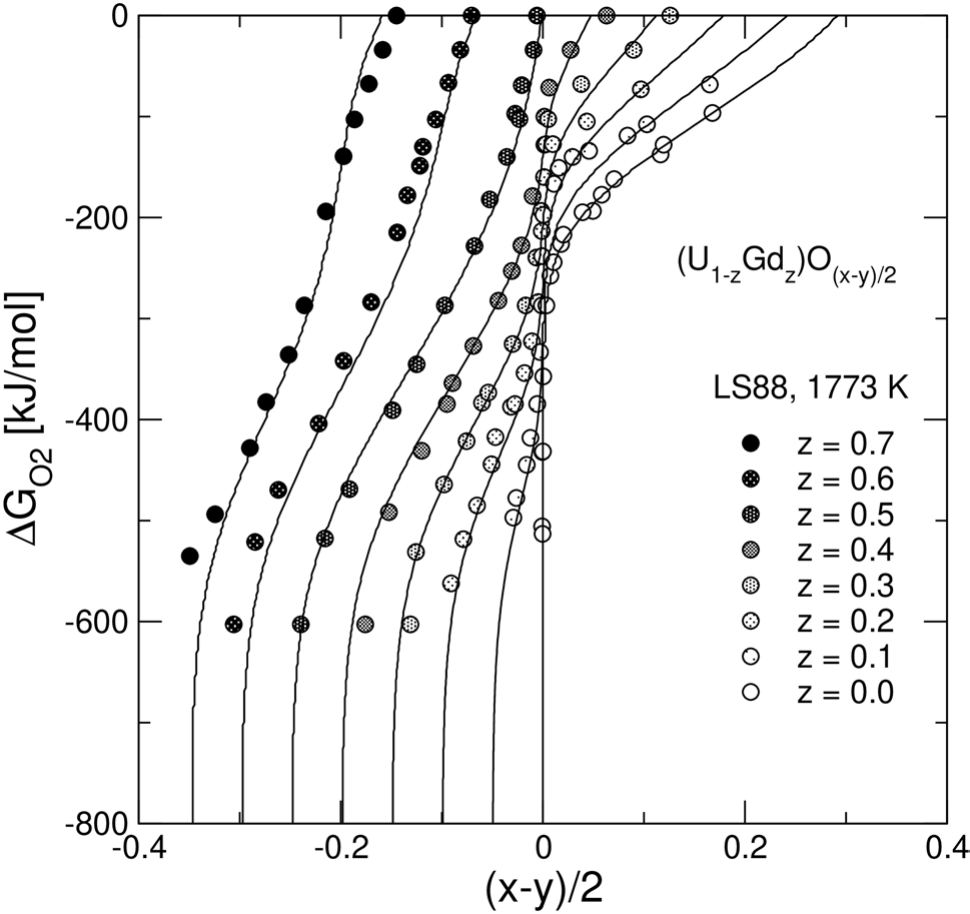
Model fit to the experimental data on ${\Delta G}_{{\mathrm{O}}_{2}}=\mathit{RT}\mathrm{ln}({P}_{{\mathrm{O}}_{2}}/{P}^{0})$ vs. $\delta =(x-y)/2$ for the system GdO_1.5_-UO_2_-UO_3_. The experimental data are from Lindemer & Sutton^36^. Note, that *δ* can be written as *δ* = (*x* − *y*)/2, as it is a function of the mole fractions, *x* and *y*, of the UO_2.5_ and *Ln*O_1.5_ endmembers. *δ* can be also evaluated as *δ* = *O/M* − 2. A negative/positive deviation from *δ* = 0 implies the presence of either oxygen vacancies or oxygen interstitials. Note also, that *δ* characterizes non-stoichiometry only of a mono-phase fluorite, while *O/M* − 2 is also applicable to a poly-phase system. ${\Delta G}_{{\mathrm{O}}_{2}}$ is a convenient function to visualize effects of the temperature and/or the partial pressure of O_2_ on the chemical potential of O_2_, while the dimensionless quantity $\mathrm{log}({P}_{{\mathrm{O}}_{2}}/{P}^{0})$ is more convenient when the temperature and the pressure effects need to be distinguished. Both quantities are used throughout the text.

The fluorite phase, U_1-*z*_*Ln*_*z*_O_2+*δ*_, is modelled here as a mixture of the endmembers UO_2_, *Ln*O_1.5_, UO_2.5_, ${{\mathrm{U}}_{1/2}{Ln}_{1/2}\mathrm{O}}_{2}$, and ${{\mathrm{U}}_{1/3}{Ln}_{2/3}\mathrm{O}}_{2}$ (see “Methods”). The model appears to be sufficiently robust to describe the dependence of the non-stoichiometry parameter, *δ*, on *T*, *z*, and ${P}_{{\mathrm{O}}_{2}}$. Figure 1 shows the model fit to the data for the pure UO_2_-UO_3_ system. Figure 2 shows the fit to the data for the GdO_1.5_-UO_2_-UO_3_ system, where the composition, *z*, is varied within the range of 0 < *z* < 0.7. The fit to ${\Delta G}_{{\mathrm{O}}_{2}}$ vs. *δ*, where ${\Delta G}_{{\mathrm{O}}_{2}}=\mathit{RT}\mathrm{ln}({P}_{{\mathrm{O}}_{2}}/{P}^{0})$; ${P}^{0}=101325\mathrm{\,Pa}$, is improved significantly relative to^32^, particularly, at *z* > 0.5. This is the result of extending the interval of *δ* to (−1/2 < *δ* < 1/3) and of including the new endmember ${{\mathrm{U}}_{1/3}{Ln}_{2/3}\mathrm{O}}_{2}$. The model parameters are given in Table 1. Importantly, some of the fitted parameters appear to be close to the values estimated from the data on UO_2_, *Ln*O_1.5_, γ-UO_3_ and U_3_O_8_ with the additivity rule (see Table S1 in Supplementary materials). This is observed, for example, in the case of UO_2.5_, but not in the cases of ${{\mathrm{U}}_{1/2}{Ln}_{1/2}\mathrm{O}}_{2}$ and ${{\mathrm{U}}_{1/3}{Ln}_{2/3}\mathrm{O}}_{2}$. The fitted standard free energies of the latter endmembers (−76.0 and −72.0 kJ/mol, respectively) appear to be significantly lower than the corresponding estimates based on additivity. This implies that these endmembers are significantly stabilized due to certain interactions occurring between UO_2.5_ and *Ln*O_1.5_ and between UO_3_ and *Ln*O_1.5_. A good fit was obtained by defining Margules parameters only for UO_2_-UO_2.5_ and UO_2_-GdO_1.5_ interactions. All other interactions were set athermal. The same model provided a reasonably good fit to ${\Delta G}_{{\mathrm{O}}_{2}}$ vs. *δ* data for systems with NdO_1.5_^33^ and LaO_1.5_^34,35^. This is illustrated in Figs. S1 and S2 (Supplementary materials). Thus, practically, the model is applicable to systems with *Ln* = {La, Nd, Gd} with no modification.

**Table 1:** Adopted thermodynamic parameters for the solid solution phases.

**Fluorite solid solution (cubic)**				
*Endmember*	*i*	$\Delta G_{i}^{0}$(kJ/mol)	$\Delta S_{i}^{0}$(J/K/mol)	$\Delta {Cp}_{i}^{0}$(J/K/mol)
${\mathrm{UO}}_{2}$	1	0.0	0.0	0.0
${Ln\mathrm{O}}_{1.5}$	2	0.0	0.0	0.0
${\mathrm{UO}}_{2.5}$	3	−81.025	7.0	8.5
${Ln}_{1/2}^{3}{{\mathrm{U}}_{1/2}^{5}\mathrm{O}}_{2}$	4	−76.0	0.0	4.25
${Ln}_{2/3}^{3}{{\mathrm{U}}_{1/3}^{6}\mathrm{O}}_{2}$	5	−72.0	3.5	6.0
*Margules parameter*	*i,j*	${W}_{ij}^{h}$(kJ/mol)	${W}_{ij}^{s}$(J/K/mol)	
	1,2	28.0	0.0	
	1,3	28.3	27.75	
**U**_**4**_**O**_**9**_ **solid solutions (cubic, β or γ)**				
*Endmember*	*i*	${\Delta G}_{i}^{0}$(kJ/mol)	${\Delta S}_{i}^{0}$(J/K/mol)	${\Delta Cp}_{i}^{0}$(J/K/mol)
${\mathrm{U}}_{1/2}^{4}{{\mathrm{U}}_{1/2}^{5}\mathrm{O}}_{9/4}$	1	−37.75	8.7	6.0
$\{{{Ln}_{1/4}^{3}\mathrm{U}}_{1/4}^{5}{{\}\mathrm{U}}_{1/2}^{5}\mathrm{O}}_{9/4}$	2	−80.3	6.37	3.0
*Margules parameter*	*i,j*	${W}_{ij}^{h}$(kJ/mol)	${W}_{ij}^{s}$(J/K/mol)	
	1,2	0.0	0.0	
**α-U**_**3**_**O**_**8**_ **solid solution (orthorhombic)**				
*Endmember*	*i*	${\Delta G}_{i}^{0}$(kJ/mol)	${\Delta S}_{i}^{0}$(J/K/mol)	${\Delta Cp}_{i}^{0}$(J/K/mol)
${\mathrm{U}}_{2/3}^{5}{{\mathrm{U}}_{1/3}^{6}\mathrm{O}}_{8/3}$	1	−95.3	11.21	15.7
$\{{{Ln}_{2/9}^{3}\mathrm{U}}_{4/9}^{6}{{\}\mathrm{U}}_{1/3}^{6}\mathrm{O}}_{8/3}$	2	−97.7	14.84	14.05
*Margules parameter*	*i,j*	${W}_{ij}^{h}$(kJ/mol)	${W}_{ij}^{s}$(J/K/mol)	
	1,2	5.0	0.0	
**α’-U**_**3**_**O**_**8**_ **solid solution (hexagonal)**				
*Endmember*	*i*	${\Delta G}_{i}^{0}$(kJ/mol)	${\Delta S}_{i}^{0}$(J/K/mol)	${\Delta Cp}_{i}^{0}$(J/K/mol)
${\{\mathrm{U}}_{2/3}^{5}{{\mathrm{U}}_{1/3}^{6}\}\mathrm{O}}_{8/3}$	1	−92.0	11.21	15.7
$\{{{Ln}_{2/9}^{3}\mathrm{U}}_{7/9}^{6}{\}\mathrm{O}}_{8/3}$	2	−97.7	14.84	14.05
*Margules parameter*	*i,j*	${W}_{ij}^{h}$(kJ/mol)	${W}_{ij}^{s}$(J/K/mol)	
	1,2	5.0	0.0	
**A-*Ln***_**2**_**O**_**3**_ **(hexagonal)**				
${Ln\mathrm{O}}_{1.5}$	1	3.3	7.5	0.0

Cations taken in curly brackets contribute to the configurational entropy of an endmember. This contribution is not included in the standard entropy values of the endmembers given in the Table but is counted within the general equation describing the entropy of mixing of a relevant phase. Upper symbols in structural formulas denote charges of cations.

### Oxidation of pure UO_2_

**Figure 3:**
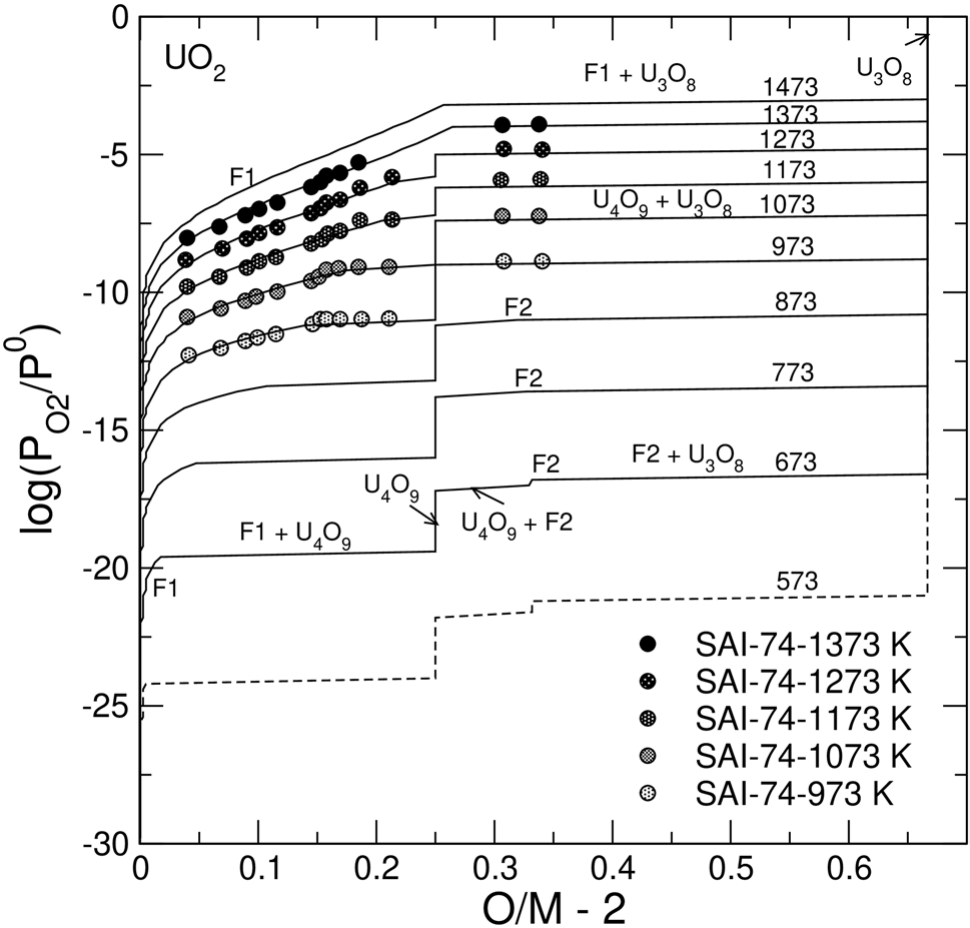
Model fit to the experimental data on $\mathrm{log}({P}_{{\mathrm{O}}_{2}}/{P}^{0})$ vs. $O/M-2$ for pure UO_2_. The experimental data are from Saito ^38^. Solid lines correspond to the equilibrium with the hexagonal polymorph of U_3_O_8_. The dashed line corresponds to the orthorhombic α-U_3_O_8_.

The equilibrium oxidation of pure UO_2_ at 973–1273 K^38,39^ occurs through the following sequence of transformations. First, the *O/M* ratio increases within the mono-phase fluorite, F, reaching a certain limiting value, then, a two-phase F + U_4_O_9_ mixture develops. After the fluorite phase in the mixture becomes extinct, a mono-phase U_4_O_9_ remains stable within a certain interval of $\mathrm{log}({P}_{{\mathrm{O}}_{2}}/{P}^{0})$, and, finally, a two-phase assemblage with varying fractions of U_4_O_9_ and U_3_O_8_ evolves, reaching an O/M ratio of ~ 2.6. The sequence F → F + U_4_O_9_ → U_4_O_9_ → U_4_O_9_ + U_3_O_8_ → U_3_O_8_ is successfully reproduced in Fig. 3. The construction of the diagram required defining the standard Gibbs free energies of the endmembers. ${\mathrm{U}}_{1/2}^{4}{{\mathrm{U}}_{1/2}^{5}\mathrm{O}}_{9/4}$ and ${\{\mathrm{U}}_{2/3}^{5}{{\mathrm{U}}_{1/3}^{6}\}\mathrm{O}}_{8/3}$ that correspond to γ-U_4_O_9_ and to the hexagonal α’-U_3_O_8_ polymorphs, respectively. At temperatures below 873 K the model predicts a reappearance of the fluorite phase, F2, with *δ* ~ 0.33, which at *O/M* > 2.33 co-exists with U_3_O_8_. The sequence of transformations below 873 K is thus F1 → F1 + U_4_O_9_ → U_4_O_9_ → U_4_O_9_ + F2 → F2 → F2 + U_3_O_8_ → U_3_O_8_. The symbols F1 and F2 distinguish different states within the same fluorite phase. The transformation U_4_O_9_ → F2 is driven by an increase in the chemical potential of oxygen caused by the decrease in temperature. The stabilization of F2 fluorite with *δ* ~ 0.33 and the disappearance of U_4_O_9_ (*δ* = 0.25) is the consequence of a larger *δ* value achievable in fluorite. The model allows to qualitatively explain the experimentally observed appearance of fluorite-like tetragonal phases with *δ* ~ 0.33 at *T* < 823^31^. Although the tetragonal β-U_3_O_7_ phase differs structurally from fluorite^40^, its thermodynamic properties are likely similar to F2 fluorite with *δ* ~ 0.33. Indeed, the data of Grenthe et al.^41^ indicate that U_3_O_7_ is stable just by 0.7 ± 2.0 kJ/mol relative to the mixture of UO_2.25_ and UO_2.67_. Similarly, in our simulations, F2 fluorite (*δ* ~ 0.33) is marginally stable relative to the same phases at temperatures below 873 K. At ~ 1373 K U_4_O_9_ becomes unstable relative to the F1 phase (with *δ* ~ 0.25). Thus, above 1373 K the phase sequence simplifies to F1 → F1 + U_3_O_8_.

### Oxidation of Ln-doped fluorite

**Figure 4:**
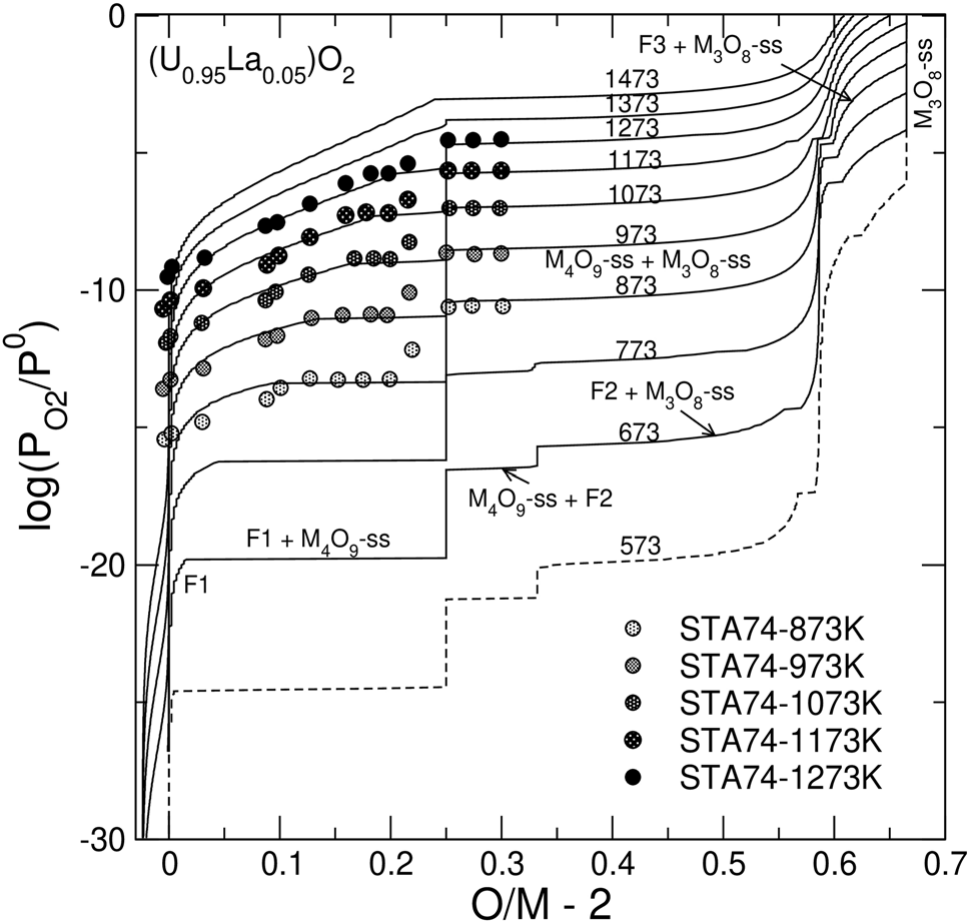
Model fit to the experimental data on $\mathrm{log}({P}_{{\mathrm{O}}_{2}}/{P}^{0})$ vs. $O/M -2$ for UO_2_ doped with 4.8 mol % of LaO_1.5_. The experimental data are from Stadlbauer et al. ^29^. Solid lines correspond to the equilibrium with the hexagonal polymorph of M_3_O_8_. The dashed line corresponds to the orthorhombic α-M_3_O_8_.

**Figure 5:**
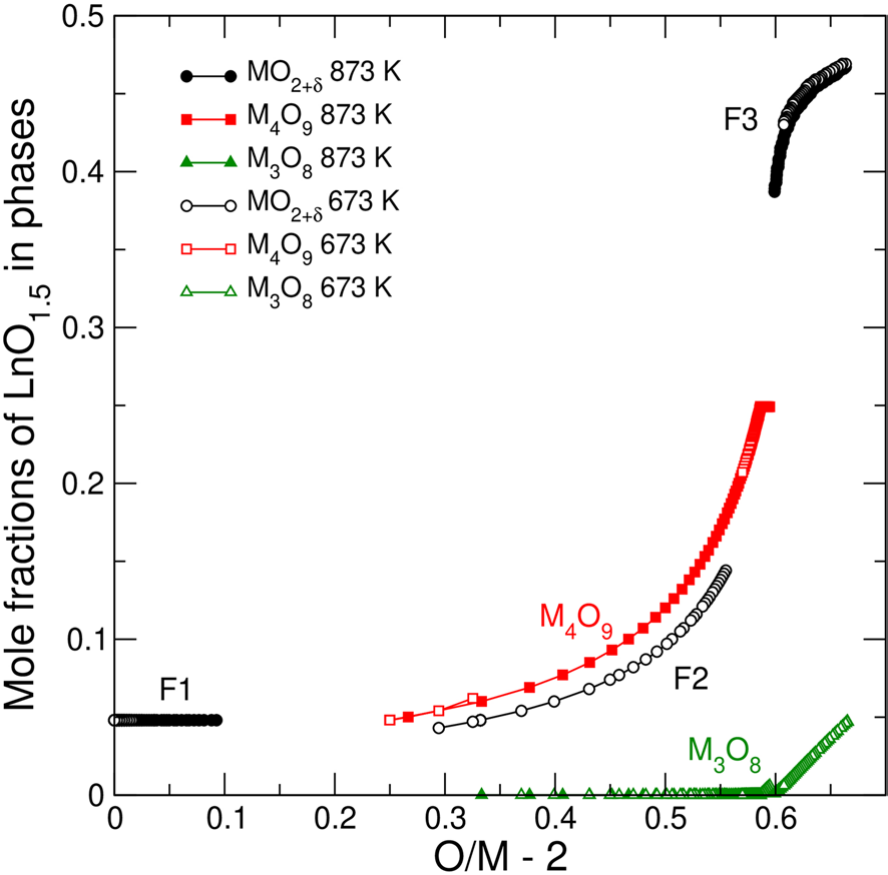
Predicted evolution of composition of phases in a sample containing 4.8 mol % of LaO_1.5_ in the process of equilibrium oxidation at 673 and 873 K. Filled and empty symbols correspond to 873 K and 673 K isotherms, respectively.

**Figure 6:**
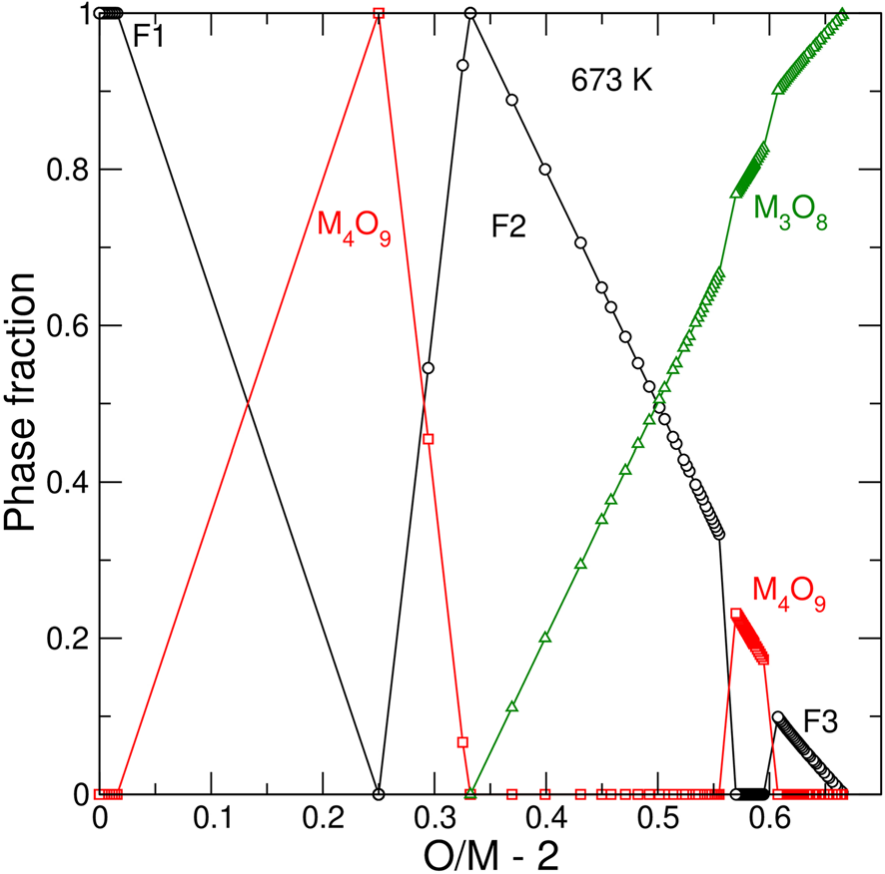
Fractions of phases in a sample containing 4.8 mol % of LaO_1.5_ in the process of equilibrium oxidation at 673 K. Symbols are the same as in Fig. 5.

Figure 4 shows the fit to the data of Stadlbauer et al.^29^ for UO_2_ doped with 4.8 mol % of LaO_1.5_. The fit required defining the standard Gibbs free energies of the endmembers $\{{{Ln}_{1/4}^{3}U}_{1/4}^{5}{{\}\mathrm{U}}_{1/2}^{5}\mathrm{O}}_{9/4}$ and $\{{{Ln}_{2/9}^{3}U}_{7/9}^{6}{\}\mathrm{O}}_{8/3}$ for the γ- and α’-phases, respectively. Stadlbauer et al.^29^ concluded that the phase relations in the doped system remain essentially the same as in the pure system. Thus, one would expect to see the phase sequence F1 → F1 + M_4_O_9_ → M_4_O_9_ → M_4_O_9_ + M_3_O_8_ → M_3_O_8_. However, the predicted phase relations (Fig. 4) are more complicated at high *O/M* ratios. At *O/M* ~ 2.60 fluorite reappears, and the sequence is modified as: F1 → F1 + M_4_O_9_ → M_4_O_9_ → M_4_O_9_ + M_3_O_8_ → F3 + M_3_O_8_ → M_3_O_8_. Importantly, the F3 phase appearing at *O/M* = 2.60 differs significantly from the F1 phase occurring within the range of 0 < *O/M* < 2.25. This F3 phase is very *Ln*-rich, while its *O/M* ratio is close to 2.0. Notably, the F2 phase with *δ* ~ 0.33 is also stable in the doped system at *T* < 873. The most significant difference relatively to the pure system is the rise of the isotherms within the biphasic M_4_O_9_ + M_3_O_8_, F2 + M_3_O_8_ and F3 + M_3_O_8_ regions to higher oxygen partial pressures. Figures 5 and 6 investigate the corresponding changes in detail. The positive slope of the isotherms in Fig. 4 at *O/M* > 2.25 correlates with the increase in the *Ln*-content in the M_4_O_9_ phase (Fig. 5). As the *O/M* ratio increases, *Ln* accumulates in M_4_O_9_, while M_3_O_8_ takes a negligeable part of the total *Ln*O_2_. The strong partitioning of *Ln* into M_4_O_9_ can be attributed to the stability of the $\{{{Ln}_{1/4}^{3}U}_{1/4}^{5}{{\}\mathrm{U}}_{1/2}^{5}\mathrm{O}}_{9/4}$ endmember. The assessed standard Gibbs free energy of the $\{{{Ln}_{1/4}^{3}U}_{1/4}^{5}{{\}\mathrm{U}}_{1/2}^{5}\mathrm{O}}_{9/4}$ endmember (Table 1), is approximately equal to the half sum of the free energies of UO_2.5_ and ${{\mathrm{U}}_{1/2}{Ln}_{1/2}\mathrm{O}}_{2}$. Thus, the high stability of ${{\mathrm{U}}_{1/2}{Ln}_{1/2}\mathrm{O}}_{2}$ component contributes to the stability of $\{{{Ln}_{1/4}^{3}}U_{1/4}^{5}{{\}\mathrm{U}}_{1/2}^{5}}\mathrm{O}_{9/4}$. As $\mathrm{log}({P}_{{\mathrm{O}}_{2}}/{P}^{0})$ increases, the fraction of M_3_O_8_ grows up while the fractions of M_4_O_9_ and/or F2 phases decrease (Fig. 6). This change occurs because a high total *O/M* ratio (e.g., *O/M* ~ 2.6) could only be achieved when the fraction of M_3_O_8_ is large. Because M_3_O_8_ is nearly free of *Ln* in the association with M_4_O_9_, the concentration of *Ln*O_1.5_ within the minor M_4_O_9_ phase increases with *O/M* and quite rapidly reaches the theoretical limit of *z* = 0.25. After the M_4_O_9_ and F2 phases vanish, fluorite remains as the F3 phase. The content of *Ln* in the F3 phase rapidly increases to ~ 45 mol %. This implies that the equilibrium oxidation of the doped sample containing 4.8 mol % of LaO_1.5_ requires a nine-fold enrichment of the fluorite phase in *Ln*O_1.5_.

The ratio of *O/M* ~ 2.0 within the *Ln*-rich F3 phase – the consequence of an exceptionally strong stability of the ${{\mathrm{U}}_{1/2}{Ln}_{1/2}\mathrm{O}}_{2}$ endmember –  has an important impact on phase relations at high ${P}_{{\mathrm{O}}_{2}}$. When $\mathrm{log}({P}_{{\mathrm{O}}_{2}}/{P}^{0})$ approaches zero, the partitioning of *Ln* into the M_3_O_8_ phase becomes favourable (Fig. 5). This partitioning is driven by the tendency of the system to achieve the largest total *O/M* ratio of ~ 2.67, which would be impossible to attain if the stoichiometric F3 fluorite (*δ* ~ 0.0) remained as a significant fraction in the system. The increase in the total *O/M* at *O/M* > 2.6 thus causes a decrease in the fraction of F3 fluorite in the mixture and an increase in the concentration of *Ln* in M_3_O_8_ (Figs. 5 and 6). The final state of a fully oxidized *Ln*-doped UO_2_ is a mono-phase *Ln*-doped M_3_O_8_. This prediction remains valid only at temperatures below ~ 973 K. As the temperature increases, the chemical potential of oxygen decreases, and the final assemblage is biphasic, consisting of an *Ln*-poor M_3_O_8_ and a very *Ln*-rich nearly stoichiometric fluorite. Figures S3 and S4 (Supplementary materials) illustrate the equilibrium oxidation at 10 mol % of *Ln*O_1.5_.

### Lattice parameter variation as a function of oxygen partial pressure, temperature, and Ln-concentration

**Figure 7:**
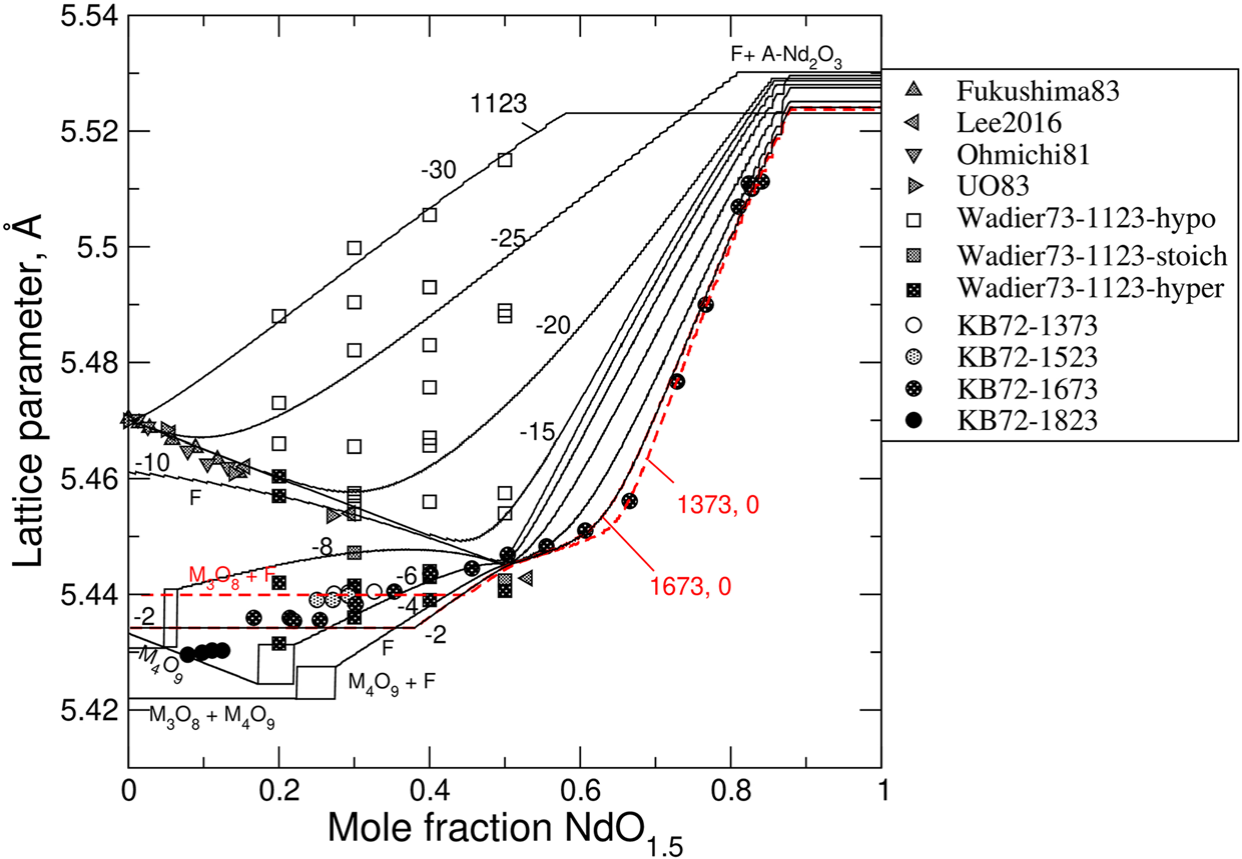
Variation of the lattice parameter in UO_2_-NdO_1.5_ solid solutions predicted from the thermodynamic model. Solid lines are the 1123 K isotherms computed at different values of $\mathrm{log}({P}_{{\mathrm{O}}_{2}}/{P}^{0})$ such that the pressure variation approximately covers the range of redox conditions in the data of Wadier ^45^. Dashed lines (red online) are the 1373 and 1673 K isotherms computed at $\mathrm{log}\left({P}_{{\mathrm{O}}_{2}}/{P}^{0}\right)=0$. These isotherms correspond to the synthesis conditions in the study of Keller & Boroujerdi^26^. The other experimental data are from Lee et al.^46^, Fukushima et al.^47^, Ohmichi et al.^48^ and Une & Oguma^33^.

The lattice parameter, *a*, of fluorite depends on *z* and on *δ*. This dependence is modelled here via an ion-packing model which is linked to the thermodynamic model. Figure 7 plots the equilibrium values of *a* in the system of NdO_1.5_-UO_2_-UO_3_ computed at 1123 K and at $\mathrm{log}\left({P}_{{\mathrm{O}}_{2}}/{P}^{0}\right)$ varying in the range [–30, –2]. Additionally, the isotherms of 1373 K and 1673 K are plotted at ${P}_{{\mathrm{O}}_{2}}={P}^{0}$. Hyper-, hypo- and strictly stoichiometric states can be distinguished. Flat regions in the predicted variation of *a* vs. *z* correspond to two-phase assemblages, where fluorite co-exists with M_3_O_8_ or A-Nd_2_O_3_ phases. The two-phase co-existence of fluorite and M_4_O_9_ is represented as a rectangle. The diagram predicts that M_4_O_9_ phase is stable at 1123 K within the interval of $-8<\mathrm{log}\left({P}_{{\mathrm{O}}_{2}}/{P}^{0}\right)<-4$. Thus, within this interval the phase sequence with the increase in *z* is as follows: M_3_O_8_ + M_4_O_9_ → M_4_O_9_ + F → F → F + A-Nd_2_O_3_. At a higher ${P}_{{\mathrm{O}}_{2}}$, the sequence is M_3_O_8_ + F → F → F + A-Nd_2_O_3_. An interesting feature is the change in the slope in the *a* vs. *z* relationship occurring in oxidized samples at *z* ~ 0.67. In strongly oxidized samples (i.e., at $\mathrm{log}\left({P}_{{\mathrm{O}}_{2}}/{P}^{0}\right)\sim 0, T=1123 \mathrm{\,K})$, the change of slope correlates with a rapid ingrowth in the fraction of the *Ln*O_1.5_ component, i.e., with the fraction of vacancies. Thus, below and above the composition of *z* = 0.67 the fluorite phase is markedly different; within the interval of 0.5 < *z* < 0.67 fluorite closely maintains the stoichiometric relationship (*O/M* = 2) – a feature, which reflects the stability of the endmembers ${{\mathrm{U}}_{1/2}{Ln}_{1/2}\mathrm{O}}_{2}$ and ${{\mathrm{U}}_{1/3}{Ln}_{2/3}\mathrm{O}}_{2}$, while at *z* > 0.67 it is hypo-stoichiometric. A similar change in the slope of the *a* vs. *z* relationship is also seen in the systems with La, Gd, Eu, and Y^25,27,42,43^. The dependence of the lattice parameter in the system of LaO_1.5_-UO_2_-UO_3_ is shown in Figure S5. Indeed, graphs analogous to Fig. 7 or Figure S5 can be easily constructed with the presently developed model for any *Ln*O_1.5_-UO_2_-UO_3_ system for which the ionic radii of *Ln* in 6-, 7-, and 8-fold coordination are known. Table S2 lists the relevant radii for systems with La, Nd, Gd and Y. The transformation at *z* ~ 0.67 suggests that in any oxidized *Ln*O_1.5_-UO_2_-UO_3_ system, where *Ln* is in +3 state, the solid composition at *z* ~ 0.67 is given by the formula ${{\mathrm{U}}_{1/3}^{{6,8}}{Ln}_{2/3}^{{3,8}}}\mathrm{O}_{2}$. This observation allows determining the ionic radius of an eightfold coordinated U^+6^ from $a=\frac{4}{\sqrt{3}}\left({(2/3)R}_{Ln}^{\mathrm{3,8}}+{(1/3)R}_{\mathrm{U}}^{\mathrm{6,8}}+{R}_{\mathrm{O}}\right)$ (see “Methods”). The value of ${R}_{\mathrm{U}}^{\mathrm{6,8}}$= 0.755 Å fitted to this relationship appears to be considerably smaller than the Shannon’s value of 0.86^44^. This discrepancy could possibly mean that the Shannon’s value corresponds to an uranyl-like environment, while the smaller value reflects the ionic radius of U^+6^ in the cubic coordination.

## Discussion

The remarkable thermodynamic stability of compounds with the composition of U_0.5_*Ln*_0.5_O_2_ has been previously reported based on solution calorimetry data^23,24^. The formation energies of U_1-*z*_*Ln*_*z*_O_2_ (*Ln* = Y, Nd, La) relative to stable oxides (UO_2_, UO_3_ and *Ln*O_1.5_) fall onto a linear trend Δ*H*_f,ox_ = – (103.8 ± 4.3)*z* kJ/mol^24^. Using the values of ${\Delta G}_{\mathrm{U}_{1/2}\mathrm{Ln_{1/2}}\mathrm{O_{2}}}^{0}=$ –76.0 kJ/mol, ${\Delta G}_{\mathrm{LnO}_{1.5}}^{0}$ = 3.3 kJ/mol (Table 1) and ${\Delta G}_{\mathrm{UO_{3}}}^{0}=$ –113.9 kJ/mol (Table S1 in Supplementary materials) we obtain for *Ln* = {La, Nd}: Δ*G* ~ Δ*H* = (–76–(0/4–113.9/4 + 3.3/2)) = –49.2 kJ/mol, which agrees well with the solution calorimetry trend at *z* = 0.5, as well as with ab initio calculations^49^. Considering the large endothermic formation energy of ${{\mathrm{U}}_{1/2}{Ln}_{1/2}\mathrm{O}}_{2}$ and ${{\mathrm{U}}_{1/3}{Ln}_{2/3}\mathrm{O}}_{2}$ endmembers, the tendency of samples with 0.5 < *z* < 0.67 to preserve stoichiometry in oxidizing conditions is well understood. The *O/M* = 2 appears to represent the maximum oxidation state of fluorite at *z* = 0.5. A further oxidation of the U_0.5_*Ln*_0.5_O_2_ compound to hyper-stoichiometry would require the oxidation of U^+5^ to U^+6^, which would be associated with a significant lattice contraction. Such an oxidation/contraction is not observed experimentally, suggesting that U^+6^ might be too small to be easily accommodated into the fluorite lattice. On the other hand, when an excess of *Ln*O_1.5_ over the U_0.5_*Ln*_0.5_O_2_ composition is added to fluorite, the advantage of a further annihilation of vacancies makes the U^+5^ → U^+6^ oxidation favourable; the system gains an equivalent amount of oxygen that is immediately consumed in the endothermic vacancy annihilation process. Consequently, the stoichiometric states extend to *z* = 0.67. This suggests that U^+6^ can be stable in fluorite only at an excess of *Ln*O_1.5_, i.e., when *z* > 0.5. A rapid increase in the lattice parameter at *z* > 0.67 can be attributed to the fact that at *z* > 0.67 oxygen vacancies cannot be avoided. The significantly larger effective size of the oxygen vacancy (see “Methods”) relative to the ionic radius of oxygen explains the positive slope.

The tendency of *Ln*-doped UO_2_ to keep *O/M* ~ 2 is also seen in Fig. 7 as the clustering in *a* vs. *z* data along the linear trend extending from stoichiometric UO_2_ towards fully oxidized samples with *z* ~ 0.5. In Fig. 7 this stoichiometric trend is outlined as an attractor for 1123 K isochores computed at different partial pressures of oxygen. The convergence of isochores implies the stability of stoichiometric states within a wide interval of $\mathrm{log}({P}_{{\mathrm{O}}_{2}}/{P}^{0})$. Figure S6 illustrates the evolution in the endmember fractions at 1273 K along the transition from reduced to oxidized samples. This evolution clearly shows the growing importance of the ${{\mathrm{U}}_{1/2}{Ln}_{1/2}\mathrm{O}}_{2}$ and ${{\mathrm{U}}_{1/3}{Ln}_{2/3}\mathrm{O}}_{2}$ components both with an increase in $\mathrm{log}\left({P}_{{\mathrm{O}}_{2}}/{P}^{0}\right)$ and with an increase in the mole fraction of *Ln*O_1.5_.

Consistently with the synthesis studies performed in oxygen or in air at 1173 < *T* < 1823 K^25,26^, the present model predicts a co-existence of U_3_O_8_ with a fluorite phase, where the latter is significantly enriched in *Ln*O_1.5_ (Fig. 7). This fluorite phase is predicted to be closely stoichiometric. The M_4_O_9_ phase is stable in association with M_3_O_8_ only within a narrow interval of $\mathrm{log}\left({P}_{{\mathrm{O}}_{2}}/{P}^{0}\right)$. At highly oxidizing conditions M_4_O_9_ is destabilized relative to the fluorite (F3) phase. The reason for the destabilization is the partitioning of *Ln* into M_4_O_9_ coupled with the limited ability this phase to incorporate *Ln*^+3^. Due to the same reason the M_4_O_9_ phase becomes unstable relative to fluorite even at a very small total *Ln*O_1.5_ fraction when the total *O/M* ratio is large (Figs. 5 and 6). Our study predicts, however, that at an excessively high chemical potential of O_2_ (e.g., in air at temperatures below 973 K) the association of F + M_3_O_8_ becomes unstable relative to a mono-phase M_3_O_8_ system (Figs. 5 and 6). The reason for this instability is the tendency of the *Ln*-rich fluorite to be stoichiometric. Thus, to achieve a total *O/M* ~ 2.67, the system must get rid of fluorite completely. This prediction is consistent with the results of the recent study of Potts et al.^50^.

The comparison of Figs. 3 and 4 shows that in the doped case a higher oxygen pressure is required to oxidize the system to the same *O/M* ratio, i.e., the doped system is more stable against oxidation. However, within the range of 2.25 < *O/M* < 2.5, where the main retardation effect is thought to take place, the difference in $\mathrm{log}({P}_{{\mathrm{O}}_{2}}/{P}^{0})$ is less than one unit. Thus, it is unlikely that the oxidation resistance is a pure thermodynamic effect. Kinetic factors should be given a closer attention. The model predicts that at ~ 873 K in the doped system the equilibrium transformation goes along the sequence F1 → F1 + M_4_O_9_ → M_4_O_9_ → M_4_O_9_ + M_3_O_8_ → F3 + M_3_O_8_ → M_3_O_8_, which includes several biphasic states. Figure 5 shows that these states involve partitioning of *Ln* between the phases, which is particularly strong in the cases of M_4_O_9_ + M_3_O_8_ and F3 + M_3_O_8_ co-existence. This partitioning implies that nucleation and growth of a nearly pure U_3_O_8_ from an *Ln*-bearing M_4_O_9_ requires redistributing *Ln*O_1.5_ back into the M_4_O_9_ phase. At *T* < 873 K the sequence of phases is modified to F1 → F1 + M_4_O_9_ → M_4_O_9_ → M_4_O_9_ + F2 → F2 → F2 + M_3_O_8_ → F3 + M_3_O_8_ → M_3_O_8_. The predicted stabilization of F2 in the doped system is important because this phase can possibly emulate the appearance of γ-M_4_O_9_ described by Thomas et al.^14^. It is conceivable that at a low temperature the redistribution of *Ln* between the phases would be controlled by the speed of solid-state *Ln*-diffusion. Likely, a crystallization of a small amount of U_3_O_8_ would be concurrent with the formation of a thin *Ln*-enriched layer in M_4_O_9_ or in γ-M_4_O_9_, which would have protective properties; a continuing growth of U_3_O_8_ would require a further enrichment of this layer making it less compositionally suitable for re-crystallizing into a *Ln*-poor U_3_O_8_. Even at a low doping level such a mechanism would eventually cause the formation of a layer sufficiently enriched in *Ln*, from which the growth of *Ln*-poor U_3_O_8_ would be practically impossible. In this respect a pure system differs remarkably from any doped system. The oxidation of pure UO_2_ requires oxygen diffusion only, which is known to be several orders of a magnitude faster than U self-diffusion in UO_2_^51^. On the other hand, a recent computational study^52^ showed that diffusion coefficients of U^+4^, La^+3^ and Y^+3^ differ only within an order of a magnitude, meaning that the *Ln* diffusion in UO_2_ is many orders of magnitude slower than O-diffusion. This proposition could explain a surprisingly large effect of small loads of trivalent dopants on rates of UO_2_ oxidation in air. The idea is that the resistance occurs not because the doping hinders the ability of fluorite and/or M_4_O_9_ to sorb oxygen, but because a dopant that forms a stable solid solution within fluorite or M_4_O_9_, cannot be easily transferred into a relatively *Ln*-poor more oxidised phase, i.e., U_3_O_8_, and must diffuse back into the parent cubic phase.

The proposed retardation mechanism is linked to the thermodynamic tendency of the cubic phases to be enriched in *Ln* relatively to orthorhombic or hexagonal U_3_O_8_. As discussed above, at a very high oxygen potential this partitioning scheme becomes less polarized, i.e., the concentration of *Ln *increases in the M_3_O_8_ phase, while the fraction of *Ln*-rich fluorite decreases in the system (Figs. 5 and 6). As oxidation experiments are typically performed at low temperatures in air where the *Ln*-rich M_3_O_8_ becomes stable, the proposed retardation mechanism involving *Ln* diffusion in M_4_O_9_ could be questioned. However, considering that the experiments are carried out with an excess of a preliminary reduced material, the effective partial pressure of oxygen at the reaction front should be significantly lower than the one in air. Likely, the oxygen pressure is buffered by the M_4_O_9_/M_3_O_8_ co-existence.

The retardation mechanism is discussed here mostly in the context of MO_2_ oxidation in air. An adequate description of oxidative dissolution would require the modelling of an aqueous phase containing dissolved forms of U^+6^. We could speculate, however, that an oxidation in an aqueous system would similarly include a dopant partitioning between an aqueous phase and an M_4_O_9_-like phase. A recent oxidative dissolution study of an unirradiated homogeneous mixed oxide fuel (MOX) sample with the composition of (Pu_0.27_U_0.73_)O_2_^53^ showed that the dissolution is incongruent, i.e., the ratio of the total dissolved Pu to the total dissolved U is about two orders of a magnitude lower than the Pu/U ratio in the solid, while the surface of the sample is enriched in Pu up to the composition of (Pu_0.39_U_0.61_)O_2+*δ*_. The formation of a Th-enriched protective layer has been also inferred in an electrochemical study of oxidation in the (U_1-*z*_Th_*z*_)O_2_ system^54^. Thus, the dopant partitioning (and the diffusion of a dopant within an M_4_O_9_-like phase), as a factor, cannot be excluded in dissolution experiments too. Clearly, a further understanding of the retardation effect would benefit from detailed spectroscopic and microanalytical studies of dopant partitioning in phases that crystallize at the oxidation front.

## Methods

### Thermodynamic approach

The modelling of *Ln*O_1.5_-UO_2_-UO_3_ systems is typically done with the CALPHAD methodology where solid solutions are described with the Compound Energy Formalism (CEF)^55–57^. In CEF endmembers are generated considering all possible occupations of available sublattices by admissible species^58^. In the case of the fluorite model with four cations in the M sublattice and two anionic sublattices this procedure gives 16 endmembers, many of which are not of neutral charge. In CEF the Gibbs free energies of charged endmembers are carefully constrained such that in all equations they appear only in charge-neutral combinations^59^. Thermodynamic functions of some individual endmembers may be chosen arbitrarily and their importance in determining real solid solution properties is difficult to visualise. The present model is more transparent. We employ only neutral endmembers and formulate site occupancies as linear functions of the endmember fractions. This means, for example, that the fractions of vacancies and O^−2^ interstitials in MO_2+*δ*_ fluorite are linked to the fractions of the neutral endmembers *Ln*O_1.5_ and UO_2.5_, respectively. We also allow for the presence of mixed endmembers, such as ${Ln}_{1/2}{{\mathrm{U}}_{1/2}\mathrm{O}}_{2}$ and ${Ln}_{2/3}{{\mathrm{U}}_{1/3}\mathrm{O}}_{2}$. Such endmembers cannot be easily included in CEF. Further, we introduce short-range order (SRO) constraints; we reduce the randomness of cation and anion distribution within sublattices by limiting the space available for mixing of species to a subset of available sites. For example, when a vacancy is introduced into a fluorite MO_2_ solid solution, it is not allowed to substitute for any lattice oxygen. Rather, the oxygen-sublattice splits into two imaginary sublattices. One of them accommodates vacancies, while the other remains fully occupied by O^−2^ anions. The fraction of sites over which the O/V mixing is allowed is considered as a model parameter. Decreasing this fraction emulates entropy reduction due to the vacancy/vacancy avoidance. Finally, we allow for a complete exclusion of certain species from mixing with other species. Particularly, in the fluorite model the fraction of U^+5^ cations that is needed to balance the fraction of O^−2^ interstitials is excluded from mixing with other cations. Such an exclusion emulates local cation–anion association, causing an additional entropy reduction. The main advantage is that the number of parameters needed to be defined is decreased significantly compared to CEF.

These non-standard model features prevent us from using available Gibbs free energy minimization software. Thus, the calculations are performed with our own code. Several simplifications are adopted. The thermodynamic properties of all endmembers in all phases are defined relative to a mechanical mixture of a stoichiometric UO_2_ fluorite and a hypothetical ordered *Ln*O_1.5_ with the pyrochlore, *Ln*_2_*Ln*_2_O_6_VV, structure. The Gibbs free energies of these two endmembers are set to zero at all temperatures. The Gibbs free energy of any other endmember *i* is expressed as a function of the temperature and the partial pressure of oxygen via the equation

1 $${G}_{i}=\Delta {G}_{i}^{0}-\left(T-{T}^{0}\right)\Delta {S}_{i}^{0}+\Delta {Cp}_{i}^{0}\left(T-{T}^{0}-Tln(\frac{T}{{T}^{0}})\right)-\Delta {n}_{i}{\mu }_{\mathrm{O}_{2}}^{T,{P}_{\mathrm{O}2}}.$$

${G}_{i}$ is set equal to the Gibbs free energy change in a reaction by which an endmember is obtained from an equivalent mixture of UO_2_, *Ln*O_1.5_ (pyrochlore) and O_2_ gas. Here ${\Delta G}_{i}^{0}$, ${\Delta S}_{i}^{0}$ and $\Delta {Cp}_{i}^{0}$ are fitting parameters and ${\Delta n}_{i}$ is the number of moles of O_2_ gas consumed or added when the endmember *i* is built from a mixture of UO_2_ and *Ln*O_1.5_. For example, the endmember ${Ln}_{1/2}{{\mathrm{U}}_{1/2}\mathrm{O}}_{2}$ of the fluorite solid solution is formed via the reaction

2 $$\frac{1}{2}{\mathrm{UO}}_{2}+{\frac{1}{2}Ln\mathrm{O}}_{1.5}+\frac{1}{8} {\mathrm{O}}_{2}={{Ln}_{1/2}{\mathrm{U}}_{1/2}\mathrm{O}}_{2}.$$

Thus, for *i* = ${{Ln}_{1/2}{\mathrm{U}}_{1/2}\mathrm{O}}_{2}$ ${\Delta n}_{i}=\frac{1}{8}$ . Similarly, the endmember ${{Ln}_{1/4}{\mathrm{U}}_{3/4}\mathrm{O}}_{9/4}$ of the M_4_O_9_-type solid solution is obtained via the reaction

3 $$\frac{3}{4}{\mathrm{UO}}_{2}+{\frac{1}{4}Ln\mathrm{O}}_{1.5}+\frac{3}{16} {\mathrm{O}}_{2}={{Ln}_{1/4}{\mathrm{U}}_{3/4}\mathrm{O}}_{9/4}.$$

In this case ${\Delta n}_{i}=\frac{3}{16}$ . The chemical potential of oxygen at a given temperature and a given partial pressure of O_2_ is computed via the equation

4 $${\mu }_{{\mathrm{O}}_{2}}^{T,{P}_{\mathrm{O}2}}={-S}_{{\mathrm{O}}_{2}}^{0}\left(T-{T}^{0}\right)+{Cp}_{{\mathrm{O}}_{2}}^{0}\left(T-{T}^{0}-Tln(\frac{T}{{T}^{0}})\right)+RT\mathrm{ln}({P}_{{\mathrm{O}}_{2}}/{P}^{0}),$$

where ${S}_{{\mathrm{O}}_{2}}^{0}=$ 205.1373 J/K/mol and ${Cp}_{{\mathrm{O}}_{2}}^{0}=$ 29.355 J/K/mol and where ${P}^{0}$ is the standard pressure of 101325 Pa, ${T}^{0}=298.15 K$^60^. Practically, the Gibbs free energy of an endmember and of a phase is made dependent not only on the temperature, but also on the partial pressure of oxygen. With this simplification modelling of the gas phase is not needed as its thermodynamic effect is included in the definition of the free energies of the solids.

The Gibbs free energy of a solid solution phase is described with a model that combines features of molecular mixing and sublattice models. The reference Gibbs free energy of a phase is modelled with the equation

5 $${G}^{\mathrm{ref}}={\sum }_{i}{X}_{i}{G}_{i},$$

where ${X}_{i}$ is the endmember fraction. The endmembers as chemical components are thought to be split into molecular cation and anion entities, which are allowed to mix separately within sublattices. In this respect ${\{\mathrm{U}}^{4}$}, ${\{Ln}_{1/2}^{3}{\mathrm{U}}_{1/2}^{5}$}, ${\{Ln}_{2/3}^{3}{\mathrm{U}}_{1/3}^{6}$}, ${\{\mathrm{U}}^{5}\}$ and ${\{\mathrm{Ln}}^{3}\}$ are legitimate molecular cation entities of the fluorite phase, which can substitute each other within the cationic sublattice. The enthalpy of mixing within a sublattice is described with the regular mixing model

6 $${G}^{\mathrm{exess}}={\sum }_{j\ne i}{x}_{i}{x}_{j}\left({W}_{ij}^{\mathrm{h}}-T{W}_{ij}^{\mathrm{s}}\right),$$

where ${x}_{i}$ is the fraction of molecular entity *i* within a sublattice. In a case of a binary solid solution, such as M_4_O_9_ or M_3_O_8_, the mixing is assumed to occur only within the cationic sublattice, and the fractions of molecular species are set equal to the endmember fractions. The ${x}_{i}={X}_{i}$ relation is also obtained in the case when enthalpic interactions are set to zero in the anionic sublattice. Such a model is adopted for fluorite. The configurational entropy is built from contributions from different sublattices as in CEF. But, when computing the entropy, the molecular entities, such as ${\{Ln}_{2/3}^{3}{\mathrm{U}}_{1/3}^{6}$}, are split into their elemental constituents. The free energy of a phase is described as follows

7 $${G}^{\mathrm{mix}}={G}^{\mathrm{ref}}+{G}^{\mathrm{exess}}-T{S}^{\mathrm{conf}},$$

where the last term combines entropic contributions from all sublattices. The free energy minimization is performed via a simple grid approach, where ich parameter is varied with a small increment over the whole parameter space. Special cases are discussed below.

### Fluorite solid solution

The model of *Ln*-doped fluorite is improved relative to the previous study^32^ in two important aspects. First, an additional stoichiometric endmember, ${{\mathrm{U}}_{1/3}{Ln}_{2/3}\mathrm{O}}_{2}$ is introduced. This endmember, together with ${{\mathrm{U}}_{1/2}{Ln}_{1/2}\mathrm{O}}_{2}$, accounts for the tendency of vacancies and interstitials to annihilate, favouring the stoichiometric composition. While the ${{\mathrm{U}}_{1/2}{Ln}_{1/2}\mathrm{O}}_{2}$ endmember allows maintaining the stoichiometric relationship up to the limit of *z* = 0.5, the ${{\mathrm{U}}_{1/3}{Ln}_{2/3}\mathrm{O}}_{2}$ endmember, due to the presence of U^+6^, allows extending the stoichiometric relation (*O/M* = 2) to *z* = 2/3. Second, the hypo- and hyper-stoichiometric limits are extended to *δ* = − 0.5 and *δ* = 0.33, respectively. The upper limit is smaller than the theoretically possible value of *δ* = 0.5 (*X*_UO2.5_ = 1) because of an additional SRO constraint, which is discussed below. UO_3_ is excluded from the list of independent endmembers. This is justified by the observation that the *O/M* ratio in fluorite never exceeds 2.5.

The parameter space is built via a stepwise admixing of new components/endmembers to UO_2_ fluorite as shown in Fig S7 (Supplementary materials). First, the *Ln*O_1.5_ component is added. A completely reduced solid solution is thus built of *z* moles of *Ln*O_1.5_ and 1 *– z* moles of UO_2_ giving the general formula of U_1-*z*_*Ln*_z_O_2−0.5*z*_. Then, one mole of *Ln*O_1.5_ and one mole of UO_2_ are allowed to react with 1/8 mol of O_2_ producing two moles of the endmember ${{\mathrm{U}}_{1/2}{Ln}_{1/2}\mathrm{O}}_{2}$. If the reaction progress is denoted *r*, then the fraction of this endmember per mole of M cations is 2*r*. Then, two moles of *Ln*O_1.5_ are allowed to react with one mole of UO_2_ and with 1/6 mol of O_2_ producing three moles of the endmember ${{\mathrm{U}}_{1/3}{Ln}_{2/3}\mathrm{O}}_{2}$. If the reaction progress is denoted *d*, then the fraction of this endmember is 3*d*. The remaining fraction, *y*, of *Ln*O_1.5_ is then *y* = *z – r – 2d*, while the remaining fraction, *q*, of UO_2_ is *q* = 1 *– z – r – d*. Further, this remaining fraction *q* can oxidize to UO_2.5_ along with the reaction UO_2_ + 1/4 O_2_ = UO_2.5_. If the reaction progress is denoted *x*, the fraction of UO_2.5_ is *x*, and the rest fraction of UO_2_ is 1 *– z – r – d – x*. The non-stoichiometry parameter, *δ*, is a simple function of the fractions of the endmembers UO_2.5_ and *Ln*O_1.5_, i.e., *δ* = (*x *−* y*)/2.

The structural formula becomes ${[\mathrm{U}}_{q-x}^{4}{{][}Ln_{y}^{3}][}Ln_{r}^{3}{{{{\mathrm{U}}_{r}^{5}][}Ln_{2d}^{3}}\mathrm{U}_{d}^{6}]{[\mathrm{U}}_{x}^{5}]\mathrm{O}}_{2+0.5(x-y)}$, where the square brackets embrace species contributing to endmember fractions. With the notation “UO_2_ “ = 1, “*Ln*O_1.5_ “ = 2, “UO_2.5_ “ = 3, “${{\mathrm{U}}_{1/2}{Ln}_{1/2}\mathrm{O}}_{2}$“ = 4, “${{\mathrm{U}}_{1/3}{Ln}_{2/3}\mathrm{O}}_{2}$“ = 5, the endmember fractions are given: ${X}_{1}=1-z-r-d-x$, ${X}_{2}=z-r-2d$, ${X}_{3}=x$, ${X}_{4}=2r$, and ${X}_{5}=3d$. These fractions appear in Eqns. 5 and 6. The variables *r*, *d* and *x* are treated as variational parameters. Their equilibrium values are determined from the condition of the Gibbs free energy minimum.

The *Ln*O_1.5_ endmember is assumed to have the pyrochlore (*Ln*_2_*Ln*_2_O_6_VV) structure. In this compound the cation coordination number is 6, and ¼ of oxygen sites is vacant. These vacant sites form an (empty) BCC sublattice within the oxygen lattice of a hypothetical M_4_(O,V)_8_ fluorite. Filling in all these sites gives the stoichiometry of MO_2_, where the M cation is eightfold coordinated. A partial filling produces 6-, 7- and eightfold coordinated cations. Thus, the UO_2_ fluorite and the *Ln*O_1.5_ pyrochlore are logical endmember choices for a model in which the M cations adopt exclusively the coordination numbers 6, 7 and 8. A model with the latter constraint is more reasonable from energy grounds than, for example, a model of perfect randomness, where the M cation can adopt all coordination numbers between 0 and 8. Indeed, atomistic simulation studies have shown that vacancies in fluorite-type compounds due to the Coulombic repulsion tend avoiding each other at short near-neighbour distances^61,62^, making cation coordination numbers smaller than 6 much less probable compared to the random case. To emulate this repulsion and to exclude cation coordination numbers smaller than 6, the vacancy/oxygen mixing is restricted to a BCC sublattice consisting of a quarter of available oxygen lattice sites. As the fraction of vacancies over the whole oxygen lattice is 0.25*y*, within the sublattice it is *y* (i.e., four times larger). The entropy (per one mole of M cations) is given by the equation

8 $${S}_{\mathrm{O}/\mathrm{V}:\mathrm{F}}^{\mathrm{conf}}=-(R/2)(y\mathrm{ln}\left(y\right)+\left(1-y\right)\mathrm{ln}(1-y)).$$

Similarly, the distribution of oxygen interstitials within vacant interstitial sites is assumed to be non-random. The O_i_/V_i_ mixing is restricted to a sublattice consisting of 1/3 of the available interstitial sites. This is motivated by the observation that UO_2.33_ (U_3_O_7_) represents a fluorite-related compound with the largest *O/M* ratio. As the concentration of interstitials over the whole interstitial lattice is 0.5*x*, it is three times larger within the sublattice. Thus, the entropy contribution due to the interstitials (per one mole of M cations) becomes

9 $${S}_{\mathrm{Oi}/\mathrm{Vi}:\mathrm{F}}^{\mathrm{conf}}=-(R/3)\left(\left(\frac{3x}{2}\right)\mathrm{ln}\left(\frac{3x}{2}\right)+\left(1-\frac{3x}{2}\right)\mathrm{ln}\left(1-\frac{3x}{2}\right)\right).$$

Equation 9 limits the admissible fraction of the UO_2.5_ component to *x* = 2/3.

The cations are assumed to be randomly mixed within the M site, however, the fraction, *x*, of U^+5^ cations is excluded from mixing with other cations. These are the U^+5^ cations that balance the excess negative charge of O^−2^ interstitials. We assume that these U^+5^ cations are tightly associated to the interstitials and thus do not contribute to the configurational entropy. The other cations mix randomly with each other over the fraction 1 −* x* of M sites. The fractions of *Ln*^+3^, U^+4^, U^+5^, and U^+6^ cations that are allowed to be mixed with each other are, thus, given by *t*_1_ = *z*/(1 −* x*), *t*_2_ = (*q* − *x*)/(1 −* x*), *t*_3_ = *r*/(1 −* x*) and *t*_4_ = *d*/(1 −* x*), respectively. The entropy equation is

10 $${S}_{\mathrm{M}:\mathrm{F}}^{\mathrm{conf}}=-\left(1-x\right)R\left({t}_{1}\mathrm{ln}\left({t}_{1}\right)+{t}_{2}\mathrm{ln}\left({t}_{2}\right)+{t}_{3}\mathrm{ln}\left({t}_{3}\right)+{t}_{4}\mathrm{ln}\left({t}_{4}\right)\right).$$

The entropy model could be compared to CEF. In CEF the structural formula of fluorite would be written ${(\mathrm{M})}_{1}{(\mathrm{O})}_{3/2}{{\left(\mathrm{O},\mathrm{V}\right)}_{1/2}(\mathrm{O}/\mathrm{V})}_{1/3}$. The difference is that in the present model a certain fraction of U^+5^ is excluded from mixing with other cations affecting the configurational entropy. The enthalpy of mixing is modelled differently from CEF. We assume that the excess effects within sublattices are due to interactions between *molecular* species which could be represented by combinations of ions with different charges. In the M sublattice these species are ${\{\mathrm{U}}^{4}$}, ${\{Ln}^{3}$}, ${\{Ln}_{1/2}^{3}{\mathrm{U}}_{1/2}^{5}$}, ${\{Ln}_{2/3}^{3}{\mathrm{U}}_{1/3}^{6}$}, and ${\{\mathrm{U}}^{5}$}. In the anionic sublattices the species are O^−2^ and V. The mixing within the anionic sublattices is assumed athermal. Because of the latter assumption the model of mixing in fluorite is simply mapped onto the regular model of mixing of the five endmembers.

### M_4_O_9_ solid solution

High-temperature galvanic-cell experiments on UO_2_-LaO_1.5_^29^ and UO_2_-LuO_1.5_^63^ systems showed that up to ~ 15 mol % of *Ln*O_1.5_ could be incorporated into the ordered U_4_O_9_ phase at 1273 K. At a higher content of *Ln*O_1.5_ superlattice reflections disappeared^29^. On the other hand, according to the data of Stadlbauer et al.^29^, the lattice parameter of U_1-*z*_La_*z*_O_2.23_ samples increased linearly within the range of 0 < *z* < 0.3 showing no break at the order/disorder transition. Based on this observation, we postulate a solubility mechanism of the type 2U^+4^ = *Ln*^+3^ + U^+5^ in M_4_O_9_ assuming the endmembers ${\mathrm{U}}_{1/2}^{4}{{\mathrm{U}}_{1/2}^{5}}\mathrm{O}_{9/4}$ and $\{{{Ln}_{1/4}^{3}}U_{1/4}^{5}{{\}}\mathrm{U}_{1/2}^{5}}\mathrm{O}_{9/4}$ and a theoretical solubility limit of 25 mol % of *Ln*O_1.5_. In this model we assume the O/V arrangement within the interstitial site to be fully ordered. The configurational entropy is thus due to M cations only. As in the fluorite model, the U^+5^ cations that are needed for balancing O^−2^ interstitials are excluded from mixing with *Ln*^+3^. This exclusion implies that the mixing of *Ln*^+3^ with U cations occurs over half of the M sites. If the *Ln*/M fraction in the solid solution is *z*, the fraction of $\{{{Ln}_{1/4}^{3}U}_{1/4}^{5}{{\}\mathrm{U}}_{1/2}^{5}\mathrm{O}}_{9/4}$ endmember is 4*z*. The structural formula is written $\{{{{\mathrm{U}}_{(1-4z)/2}^{4}Ln}_{z}^{3}U}_{z}^{5}{{\}\mathrm{U}}_{1/2}^{5}\mathrm{O}}_{9/4}$, where the curly brackets unite cations that are allowed to mix with each other. The configurational entropy (per one mole of M cations) is

11 $${S}_{\mathrm{M},\upgamma }^{\mathrm{conf}}=-(R/2)(4z\mathrm{ln}\left(2z\right)+\left(1-4z\right)\mathrm{ln}(1-4z)).$$

The enthalpy of mixing is modelled using a single Margules parameter for the interaction between the endmembers ${\mathrm{U}}_{1/2}^{4}{{\mathrm{U}}_{1/2}^{5}\mathrm{O}}_{9/4}$ and $\{{{Ln}_{1/4}^{3}U}_{1/4}^{5}{{\}\mathrm{U}}_{1/2}^{5}\mathrm{O}}_{9/4}$. The standard properties of the endmembers are defined according to the Eq. 1, relative to UO_2_ fluorite and a hypothetical *Ln*O_1.5_ pyrochlore. The U_4_O_9_ phase exists in α-, β- and γ-forms. The structural transitions occur at ∼323 K (α ↔ β) and at ∼873 K (β ↔ γ)^64^. The present modelling is mostly based on data corresponding to the stability of the high-temperature γ-form. However, as crystallographic differences between β- and γ-forms are minor^64^, the developed solid solution model is thought applicable to the β-form as well.

### M_3_O_8_ solid solution

At ambient temperatures U_3_O_8_ is orthorhombic *C2mm*^65^. The phase is referred to as α-form. U^+5^ and U^+6^ cations occupy two different Wyckoff positions in the proportion 2:1. With the increase in the temperature at ~ 623 K the structure becomes hexagonal, $P\overline{6 }2m$ (α’-form), where the cations occupy a single Wyckoff position. The α/α’ transition is fully reversible. At about 1173 K the hexagonal relationship ***b/a***=$\sqrt[]{3}$ is destroyed, and the structure is again described as orthorhombic. When this high-temperature structure is slowly cooled down to room temperature, it is indexed in the *Cmcm* space group^66^ and is known as β-form. The present study is mostly concerned with applications to ambient temperatures; thus, we develop models for the α and α’ phases only. Few experimental $\mathrm{log}({P}_{{\mathrm{O}}_{2}}/{P}^{0})$ vs. *δ* data that fall into the stability field of the β-form are fitted with the model for the α’-phase. This is justified by noting that the data do not show a strong variation across the α’/β transition. To fix the α/α’ transition at ~ 623 K, we assume that the entropy of the transformation is fully due to the configurational disorder of U^+5^ and U^+6^ within the single Wyckoff position of the α’-phase, which is 5.292 J/K/mol. The standard Gibbs free energy of the α-form is therefore set to be 3.3 kJ/mol more negative than the standard Gibbs free energy of the α’-phase, while the latter is fitted to experimental data. The solid solution models are developed assuming the 3U^+5^ = *Ln*^+3^ + 2U^+6^ substitution mechanism^50,67^. The solid solution in the α’-phase is modelled as a mixture of two endmembers, ${\{\mathrm{U}}_{2/3}^{5}{{\mathrm{U}}_{1/3}^{6}\}\mathrm{O}}_{8/3}$ and $\{{{Ln}_{2/9}^{3}U}_{7/9}^{6}{\}\mathrm{O}}_{8/3}$. If the *Ln*/*M* ratio in the phase is *z*, the fraction of the endmember $\{{{Ln}_{2/9}^{3}U}_{7/9}^{6}{\}\mathrm{O}}_{8/3}$ is 9*z*/2, the structural formula is ${\{\mathrm{U}}_{2/3-3z}^{5}{{Ln}_{z}^{3}U}_{1/3+2z}^{6}{\}\mathrm{O}}_{8/3}$. The entropy is given by the equation

12 $${S}_{\mathrm{\alpha {\prime}}-\mathrm{U}3\mathrm{O}8}^{\mathrm{conf}}=-R\left(z\mathrm{ln}\left(z\right)+\left(\frac{1}{3}+2z\right)\mathrm{ln}\left(\frac{1}{3}+2z\right)+\left(\frac{2}{3}-3z\right)\mathrm{ln}\left(\frac{2}{3}-3z\right)\right).$$

In the low-temperature α-phase one Wyckoff position is fully filled by U^+6^. Thus, *Ln*-substitution is thought to occur only over 2/3 of M sites. The endmembers than have the composition of ${\mathrm{U}}_{2/3}^{5}{{\mathrm{U}}_{1/3}^{6}}\mathrm{O}_{8/3}$ and $\{{{Ln}_{2/9}^{3}}U_{4/9}^{6}{{\}\mathrm{U}}_{1/3}^{6}}\mathrm{O}_{8/3}$. If the *Ln*/*M* ratio in the phase is *z*, the fraction of the $\{{{Ln}_{2/9}^{3}}U_{4/9}^{6}{{\}}\mathrm{U}_{1/3}^{6}}\mathrm{O}_{8/3}$ endmember is 9*z*/2, the structural formula becomes ${\{\mathrm{U}}_{2/3-3z}^{5}{{Ln}_{z}^{3}}U_{2z}^{6}{{\}\mathrm{U}}_{1/3}^{6}}\mathrm{O}_{8/3}$. The configurational entropy is written as

13 $${S}_{\mathrm{\alpha }-\mathrm{U}3\mathrm{O}8}^{\mathrm{conf}}=-(2/3)R\left(\left(\frac{3z}{2}\right)\mathrm{ln}\left(\frac{3z}{2}\right)+\left(3z\right)\mathrm{ln}\left(3z\right)+\left(1-\frac{9z}{2}\right)\mathrm{ln}\left(1-\frac{9z}{2}\right)\right).$$

The enthalpy of mixing is defined by Margules parameters for the interactions between ${\{\mathrm{U}}_{2/3}^{5}{{\mathrm{U}}_{1/3}^{6}\}}\mathrm{O}_{8/3}$ and $\{{{Ln}_{2/9}^{3}}U_{7/9}^{6}{\}}\mathrm{O}_{8/3}$ in the α’-phase and between ${\mathrm{U}}_{2/3}^{5}{{\mathrm{U}}_{1/3}^{6}}\mathrm{O}_{8/3}$ and $\{{{Ln}_{2/9}^{3}}U_{4/9}^{6}{{\}\mathrm{U}}_{1/3}^{6}}\mathrm{O}_{8/3}$ in the α -phase.

### Fitting strategy

Experimental data linking *δ* to *T*, *z* and $\mathrm{log}({P}_{{\mathrm{O}}_{2}}/{P}^{0})$ are most suitable for constraining the model of the fluorite phase. Here the data on pure UO_2_-UO_3_^36–38^ are used to determine the standard Gibbs free energy of UO_2.5_ (i.e., the ${\Delta G}_{i}^{0}$, ${\Delta S}_{i}^{0}$ and $\Delta {Cp}_{i}^{0}$ terms), as well as the Margules parameters (*W*_H_ and *W*_S_) for the interaction between ${\{\mathrm{U}}^{4}$} and ${\{\mathrm{U}}^{5}$} species (equivalently between UO_2_ and UO_2.5_ endmembers). The data for the GdO_1.5_-UO_2_-UO_3_ system^36^ are used for parameterizing the dependency of *δ*, on *z*, i.e., on the fraction of *Ln*O_1.5_. The minimum parameter set allowing this description includes the ${\Delta G}_{i}^{0},{\Delta S}_{i}^{0}$ and $\Delta {Cp}_{i}^{0}$ values of the endmembers ${{\mathrm{U}}_{1/2}{Ln}_{1/2}\mathrm{O}}_{2}$ and ${{\mathrm{U}}_{1/3}{Ln}_{2/3}\mathrm{O}}_{2}$, and the Margules parameter (*W*_H_) for the interaction between ${\{\mathrm{U}}^{4}$} and ${\{Ln}^{3}$}. The initial values of all parameters were estimated from available thermodynamic data on UO_2_, γ-UO_3_ and U_3_O_8_^68^ with the additivity rule (see Table S1 in Supplementary materials). The data for UO_2_ were set to zero values, while the data for γ-UO_3_ and U_3_O_8_ were recalculated as increments over the data on UO_2_. The standard properties of *Ln*O_1.5_ (pyrochlore) were set to zero. The parameters of U_4_O_9_- and U_3_O_8_-type phases were fitted to two-phase equilibrium data. Then the ${\Delta G}_{i}^{0}$ and ${\Delta S}_{i}^{0}$ of $\{{\mathrm{U}}_{1/2}^{4}{{\}\mathrm{U}}_{1/2}^{5}\mathrm{O}}_{9/4}$ were tuned such that the U_4_O_9_/UO_2+*δ*_ transition occurred at ~ 1373 K consistently with Saito^38^. The ${\Delta G}_{i}^{0}$, ${\Delta S}_{i}^{0}$ and $\Delta {Cp}_{i}^{0}$ parameters of the endmember $\{{{Ln}_{1/4}^{3}}U_{1/4}^{5}{{\}\mathrm{U}}_{1/2}^{5}}\mathrm{O}_{9/4}$ were fitted to the data of Stadlbauer et al.^29^. The ${\Delta S}_{i}^{0}$ and $\Delta {Cp}_{i}^{0}$ parameters of the $\{{{Ln}_{2/9}^{3}}U_{7/9}^{6}{\}}\mathrm{O}_{8/3}$ endmember were estimated with the additivity rule. The ${\Delta G}_{i}^{0}$ parameter of the endmember $\{{{Ln}_{2/9}^{3}}U_{7/9}^{6}{\}}\mathrm{O}_{8/3}$ endmember and the Margules parameter for the interaction between ${\{\mathrm{U}}^{5}\}$ and $\{{{Ln}_{1/3}^{3}U}_{2/3}^{6}\}$ species were adjusted such that at *T* > 1173 K in air the model predicted a strong partitioning of *Ln*O_1.5_ into the fluorite phase, consistently with the high-temperature equilibrium data^25–30^, while at *T* < 973 K the same model predicted the destabilization of fluorite relative to a *Ln*-rich M_3_O_8_ phase, consistently with the air oxidation experiments of Potts et al.^50^.

### Ion-packing model of fluorite-type phases

The lattice parameter of doped UO_2_ fluorite varies as a function of composition, non-stoichiometry, and the type of *Ln* cation^46–48^. These variations could be adequately modelled with the aid of an ion-packing model^32,48,69^, which uses the geometrical relationship between the lattice parameter, *a*, and the sum of the average radii of cations, $\langle {R}_{\mathrm{C}}\rangle ,$ and anions,$\langle {R}_{\mathrm{A}}\rangle$

14 $$a=\frac{4}{\sqrt{3}}\left(\langle {R}_{\mathrm{C}}\rangle +\langle {R}_{\mathrm{A}}\rangle \right).$$

The coefficient $\frac{4}{\sqrt{3}}$ appears from the condition that cations and anions touch each other in [111] direction. The average radii of cations and anions are further evaluated as sums of radii of all cation and anion species weighted by their fractions in the structural formula ${\{\mathrm{U}}_{q-x}^{4}{Ln}_{z}^{3}{{{\mathrm{U}}_{r}^{5}\mathrm{U}}_{d}^{6}\}{\mathrm{U}}_{x}^{5}\{\mathrm{O}}_{2-0.5y}{\mathrm{V}}_{0.5y}\}{\mathrm{O}}_{0.5x}$. The cations taken in the curly brackets split into fractions of 6-, 7-, and 8-fold coordinated species. The latter are calculated under the assumption that vacancies occur only within a BCC sublattice of the oxygen lattice of fluorite. As the fraction of vacancies in this sublattice is *y*, and as each cation is shared by two sites of the BCC sublattice, the fractions of 6-, 7-, and eightfold coordinated species are *y*^2^, 2*y*(1 −* y*) and (1 −* y*)^2^, respectively. This means, for example, that U^+4^ cations occur in the solid solution as U^4,6^, U^4,7^, and U^4,8^ species with the fractions (*q *− *x*)*y*^2^, (*q* − *x*)2*y*(1 − *y*) and (*q* − *x*)(1 − *y*)^2^, respectively. Here and below for cation species the notation *M*^*Q*,*K*^ is used, where *Q* and *K* are the charge and the coordination number, respectively. The U^+5^ cations that are not included in the curly brackets in the structural formulae are assumed to have a coordination number larger than 8. These cations balance the excess oxygen charge and are assumed to have one or two interstitial anions in their close neighbourhood. These species is denoted with the symbol ${\mathrm{U}}^{\mathrm{5,9}}$. The average radius of an anion is composed of the contributions from the lattice oxygen O^−2^ and the oxygen vacancy

15 $$\langle {R}_{\mathrm{A}}\rangle =\left(1-0.25y\right){R}_{\mathrm{O}}+0.25y{R}_{\mathrm{V}}.$$

Following the studies of Bukaemskiy et al.^69^ and Vinograd et al.^32^, we assume that a vacancy has a defined radius that is about 12% larger than the ionic radius of oxygen. The values of ${R}_{\mathrm{O}}=1.3736$ Å and ${R}_{\mathrm{V}}=1.5410$ Å are adopted from previous studies^32,69^. The oxygen interstitials are assumed to have zero ionic radius, while their effect on the lattice parameter is included into the effective size of the ${\mathrm{U}}^{\mathrm{5,9}}$ cation. Because the lattice contracts upon the oxidation from MO_2_ to MO_2+*δ*_, the ionic radius of ${\mathrm{U}}^{\mathrm{5,9}}$ is set smaller than the radius of ${\mathrm{U}}^{\mathrm{4,8}}$. The radii of ${\mathrm{U}}^{\mathrm{4,8}}$, ${\mathrm{U}}^{\mathrm{4,7}}$, ${\mathrm{U}}^{\mathrm{4,6}}$, ${\mathrm{U}}^{\mathrm{5,8}}$, ${\mathrm{U}}^{\mathrm{5,7}}$, ${\mathrm{U}}^{\mathrm{5,6}}$, ${Ln}^{\mathrm{3,8}}$, ${Ln}^{\mathrm{3,7}},{Ln}^{\mathrm{3,6}}$ are adopted with small modifications from^32^. The radius of ${\mathrm{U}}^{\mathrm{6,8}}$ is fitted to the lattice parameter data on fully oxidised UO_2_-*Ln*O_1.5_ solid solutions with the composition of *z* ~ 0.67^25,26^ under the assumption that at these conditions *Ln* and U occur exclusively as ${Ln}^{\mathrm{3,8}}$ and ${\mathrm{U}}^{\mathrm{6,8}}.$ This assumption is justified in the Results section. The radii of ${\mathrm{U}}^{\mathrm{6,7}}$ and ${\mathrm{U}}^{\mathrm{6,6}}$ are constrained under the assumption that the radii of ${\mathrm{U}}^{Q,K}$ vary linearly with *K*, while the slope of ${R}_{\mathrm{U}}^{Q,K}$ vs. *K* is the same for *Q* = {4, 5 and 6}. The radii are given in Table S2 (Supplementary materials).

The lattice parameter of the M_4_O_9_ phase is also modelled with Eqns. 14 and 15. The structural formula of M_4_O_9_ can be written as $\{{{{\mathrm{U}}_{(1-4z)/2}^{4}}Ln_{z}^{3}}\mathrm{U}_{z}^{5}{{\}}\mathrm{U}_{1/2}^{5}}\mathrm{O}_{2 }{\mathrm{O}}_{1/4}$. There are no vacancies, thus, all cations that are embraced by curly brackets occur in eightfold coordination, while the average anion radius is equal to the ionic radius of O^−2^.

### Supplementary Information

Supplementary Information.

## Data Availability

The datasets generated during and/or analysed during the current study are available from the corresponding author on reasonable request.
