# Supplementary material for: Thermodynamic model of the oxidation of Ln-doped UO2
Source: Sci Rep. 2023 Oct 20;13:17944. doi: 10.1038/s41598-023-42616-x (PMC10589314; doi:10.1038/s41598-023-42616-x)
Supplement: Supplementary file 1 — Supplementary Information. [file 41598_2023_42616_MOESM1_ESM.docx]

Thermodynamic model of the oxidation of *Ln*-doped UO_2_

V.L. Vinograd^a,*^, A.A. Bukaemskiy^a^, G. Deissmann^a^ and G. Modolo^a^

^a^ Institute of Energy and Climate Research– Nuclear Waste Management (IEK-6), Forschungszentrum Jülich GmbH, Jülich, Germany.

Supplementary materials

The materials deposited here illustrate certain important aspects of the developed thermodynamic model that could not be included in the main text due to space constraints.

Table S1 lists the fitted parameters together with initial values estimated using available thermodynamic data [1] with the aid of the additivity rule. In these calculations the $\Delta G^{0}$,
${\Delta S}^{0}$, and ${\Delta Cp}^{0}$values of UO_2_ (fluorite) and *Ln*O_1.5_ (pyrochlore) were set to zero values, while the corresponding values for UO_2.5_ (U_4_O_9_), γ-UO_3_, UO_2.67_ (U_3_O_8_), were recalculated as increments relative to the data [1] for UO_2_ (fluorite). For example, the standard Gibbs free energy of F-UO_2.5_ – the endmember of the fluorite solid solution – was estimated as

$\Delta G_{UO2.5}^{0}$= $(3\Delta G_{UO2.67}^{0}-\Delta G_{UO3}^{0})/2$, while the standard Gibbs free energy of F-U_0.5_*Ln*_0.5_O_2_ was estimated as $\Delta G_{U0.5Ln0.5O2}^{0}$= $(\Delta G_{UO2.5}^{0}+\Delta G_{LnO1.5}^{0})/2$. In these calculations the uncertainties associated with the data for *Ln*O_1.5_ were ignored. These uncertainties include the average uncertainties in the data on stable forms of *Ln*O_1.5_ and unknown effects of phase transitions from the stable *Ln*O_1.5_ forms to *Ln*O_1.5_ (pyrochlore). These uncertainties in $\Delta G_{LnO1.5}^{0}$are possibly in the range of 3-5 kJ/mol. The important message to be read from Table S1 is that the fitted values of the endmembers that contain both U and *Ln* (the last four lines in the Table S1) appear to be significantly more negative relative to the values estimated via the additivity rule.

Table S1. Fitted and estimated thermodynamic properties of the relevant phases.

|  | $\Delta G^{0}$(fit),  kJ/mol | ${\Delta S}^{0}\left( \mathrm{fit} \right)$  J/K/mol | ${\Delta Cp}^{0}\left( \mathrm{fit} \right)$  J/K/mol | $\Delta G^{0}$(exp)  kJ/mol | $\Delta S^{0}\text{(exp)}$  J/K/mol | ${\Delta Cp}^{0}\left( \exp\right)$  J/K/mol |
| --- | --- | --- | --- | --- | --- | --- |
| F-UO_2_ | **0** | **0** | **0** | 0.0 ± 1.0 | 0.0 ± 0.2 | 0.0 ± 0.2 |
| F-LnO_1.5_ | **0** | **0** | **0** | 0.0 | 0.0 | 0.0 |
| UO_2.25_ | **-37.754** | **8.7** | **6.0** | -37.2 ± 2.7 | 6.5 ± 0.4 | 9.7 ± 0.4 |
| *γ*-UO_3_ |  |  |  | -113.9 ± 3.0 | 19.08 ± 0.6 | 18.07 ± 0.3 |
| UO_2.67_ | **-92.0** | **11.208** | **15.7** | -91.36 ± 1.8 | 11.86 ± 0.4* | 15.7 ± 0.3 |
| F-UO_2.5_ | **-81.025** | **7.0** | **8.5** | *-80.1 ± 0.9* | *8.25 ± 0.5* | *14.5 ± 0.3* |
| F-U_0.5_Ln_0.5_O_2_ | **-76.0** | **0.0** | **4.25** | *-40.05 ± 0.5* | *4.125 ± 0.3* | *7.25 ± 0.2* |
| F-U_0.33_Ln_0.67_O_2_ | **-72.0** | **3.5** | **6.0** | *-37.96 ± 1.0* | *6.36 ± 0.2* | *6.0 ± 0.1* |
| U_0.75_Ln_0.25_O_2.25_ | **-80.3** | **6.37** | **3.0** | *-61.075 ± 0.7* | *6.19 ± 0.4* | *10.9 ± 0.2* |
| U_0.778_Ln_0.222_O_2.67_ | **-97.7** | **14.84** | **14.05** | *-88.59 ± 2.3* | *14.84 ± 0.5* | *14.05 ± 0.2* |

Note: The plain numbers with error bars correspond to the original data [1], the numbers in italics correspond to the parameters estimated with the aid of the additivity rule, the numbers in bold are the parameters which were fitted and/or adopted in the present study. Gray, red and green shadings unite the endmembers of MO_2_-, M_4_O_9_- and M_3_O_8_-type solid solutions, respectively. *The ideal configurational entropy of U^+5^/U^+6^ of 5.292 J/K/mol is subtracted from the tabulated value.

Figures S1 and S2 are included here with the aim to show that the thermodynamic model of fluorite, which was fitted to $\log({P_{O_{2}}}/{P^{0}})$ vs. $\delta$ data of Lindemer & Sutton [2] for the GdO_1.5_-UO_2_-UO_3_ system, provides also a reasonably good description of similar data on LaO_1.5_-UO_2_-UO_3_ and NdO_1.5_-UO_2_-UO_3_ systems.


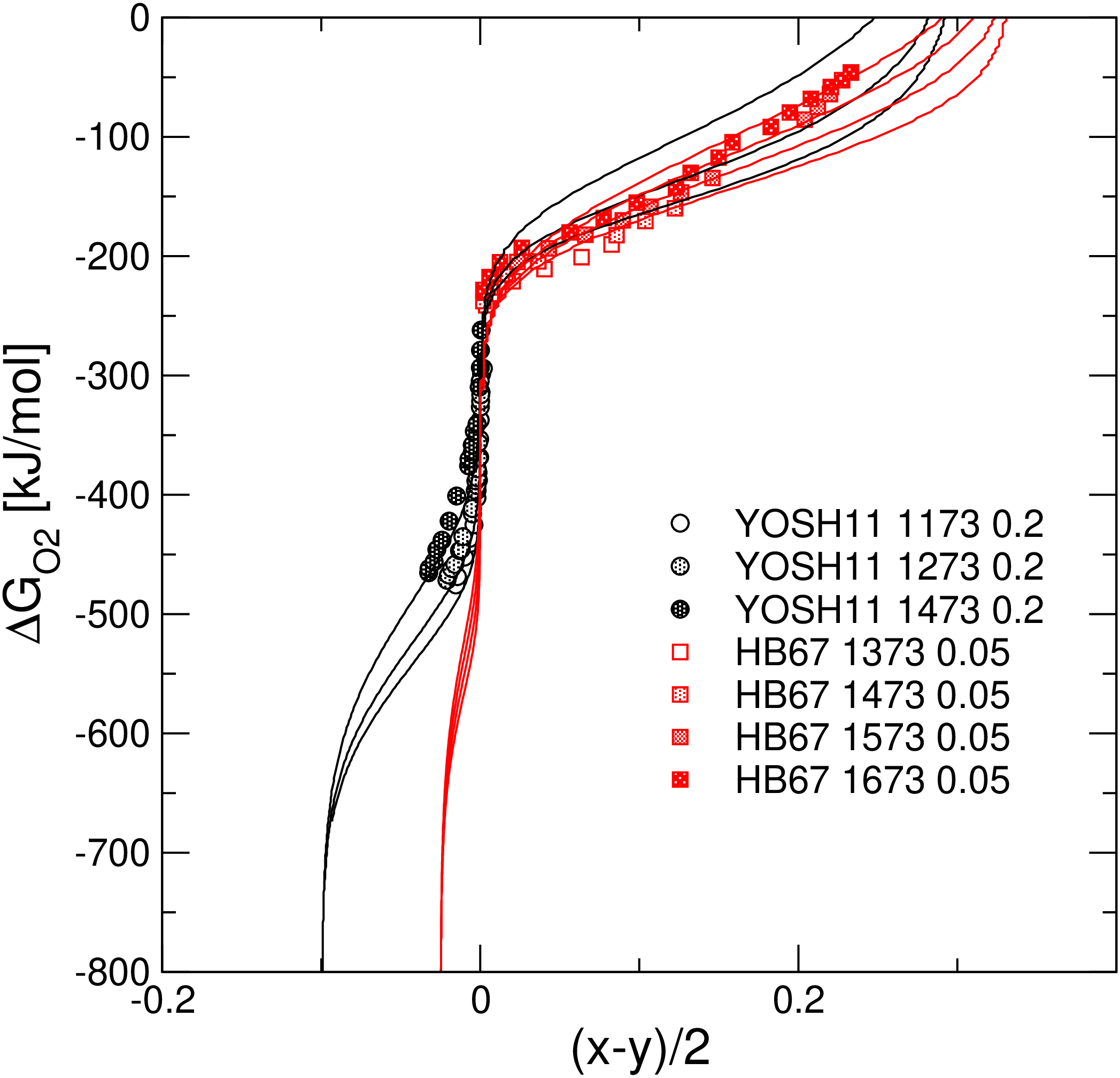


Fig. S1. Model fit to the experimental data on ${\Delta G}_{O_{2}}=RT\ln({P_{O_{2}}}/{P^{0}})$ vs. $\delta=(x-y)/2$ for the system LaO_1.5_-UO_2_-UO_3_. The experimental data are from Yoshida et al. [3] and Hagemark & Broli [4].


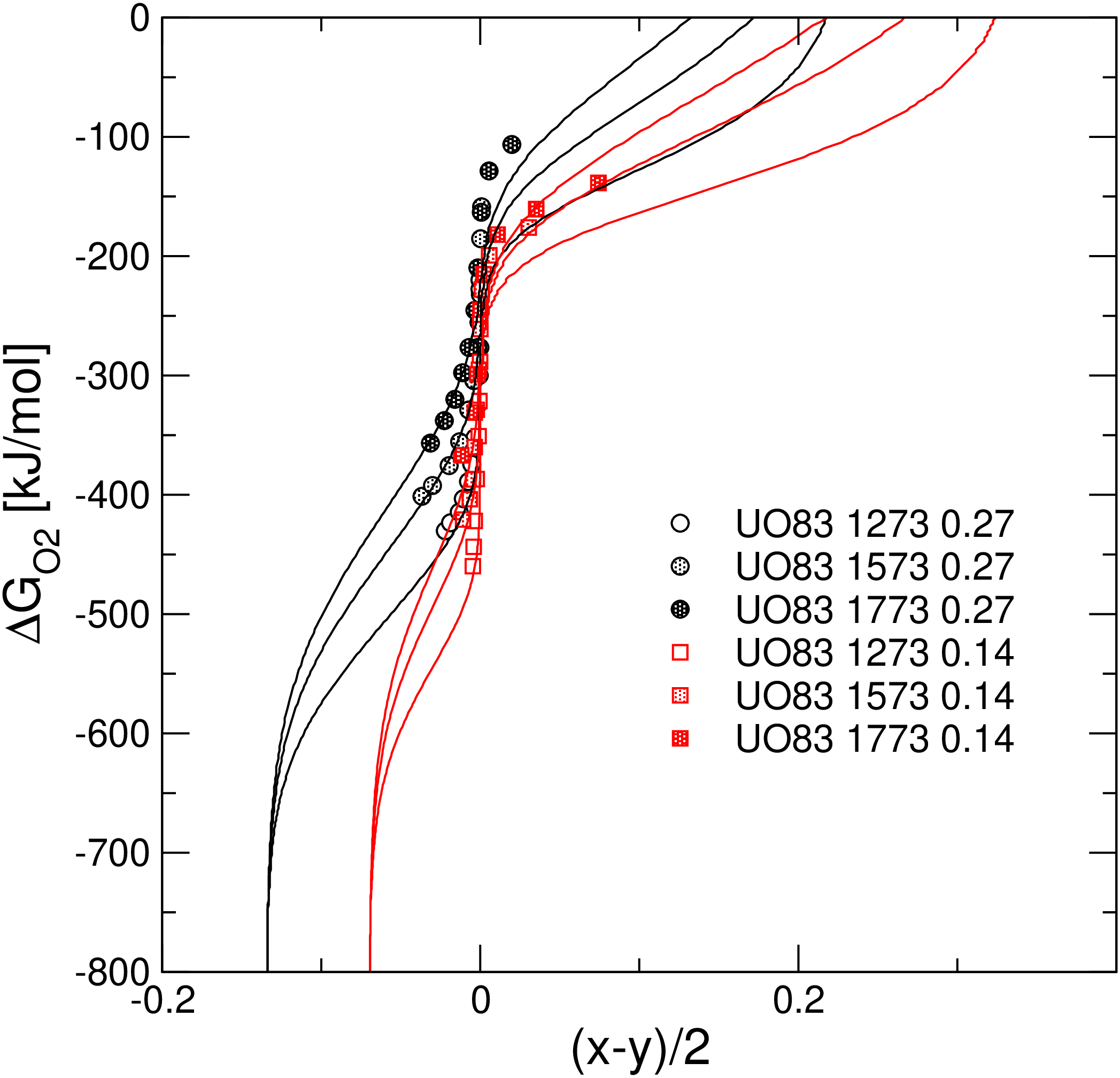


Fig. S2. Model fit to the experimental data on ${\Delta G}_{O_{2}}=RT\ln({P_{O_{2}}}/{P^{0}})$ vs. $\delta=(x-y)/2$ for the system of NdO_1.5_-UO_2_-UO_3_. The experimental data are from Une & Oguma [5].

The developed model permits predicting the equilibrium oxygen partial pressure in an *Ln*O_1.5_-UO_2_-UO_3_ system at any mole fraction of *Ln*O_1.5_. Figure S3 plots $\log\left( {P_{O_{2}}}/{P^{0}} \right)$ vs. O/M dependencies at 10 mole % of *Ln*O_1.5_. Comparing Figure S3 with Figures 3 and 4 in the main text shows that at a given partial pressure the *O/M* ratio that corresponds to the assemblage of M_4_O_9_ + M_3_O_8_ decreases drastically with the increase in the total fraction of *Ln*O_1.5_. The decrease in *O/M* is caused by the increased fraction of M_4_O_9_ (*O/M* = 2.25) in the mixture. This fraction must necessarily increase because the M_3_O_8_ is predicted to contain very little of *Ln*O_1.5_. This prediction is consistent with the experimental data of Kim et al. [6] on air oxidation of U_1-_*_z_*Gd*_z_*O_2_ within the temperature interval of 298 – 773 K. The data [6] show that the final *O/M* ratio consistently decreases with the increase in the fraction of GdO_1.5_, while the ratio of M_4_O_9_ in the final mixture increases. The observed consistency between the simulations and the experiment [6] suggests that the end points in the data of Kim et al. [6] correspond to the assemblage of M_4_O_9_ + M_3_O_8_, in which the M_3_O_8_ phase is represented by a nearly pure U_3_O_8_, while the M_4_O_9_ phase is significantly enriched in *Ln*. The equilibrium assemblage of F3 + M_3_O_8_ appears not to be reached in the experiments. The likely reason for the delay in the formation of the F3 + M_3_O_8_ assemblage is the slow diffusion of *Ln* that prevents the strong *Ln*-partitioning into the F3 phase (Fig. S4). A sample containing 10 mole% of *Ln*O_1.5_ is predicted to evolve to O/M ~ 2.5 (Fig. S4). This value is consistent with the data of Kim et al. [6] for the sample containing 9.2 mole% of GdO_1.5_.


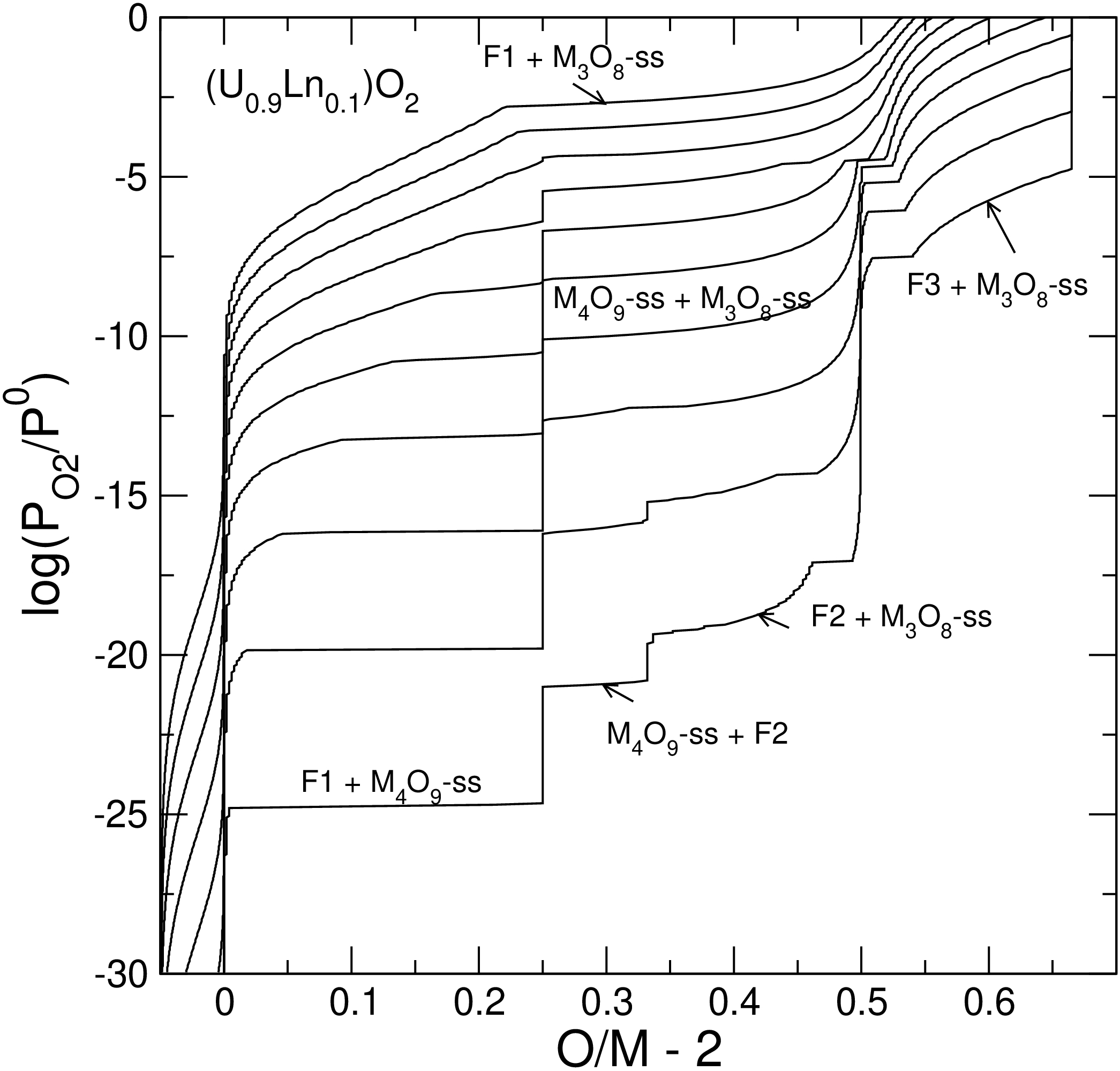


Fig. S3. Predicted variation of $\log({P_{O_{2}}}/{P^{0}})$ vs. *O/M* – 2 in UO_2_ doped with 10 mole % of *Ln*O_1.5_. Dashed lines correspond to 4.8 mole % of *Ln*O_1.5_.


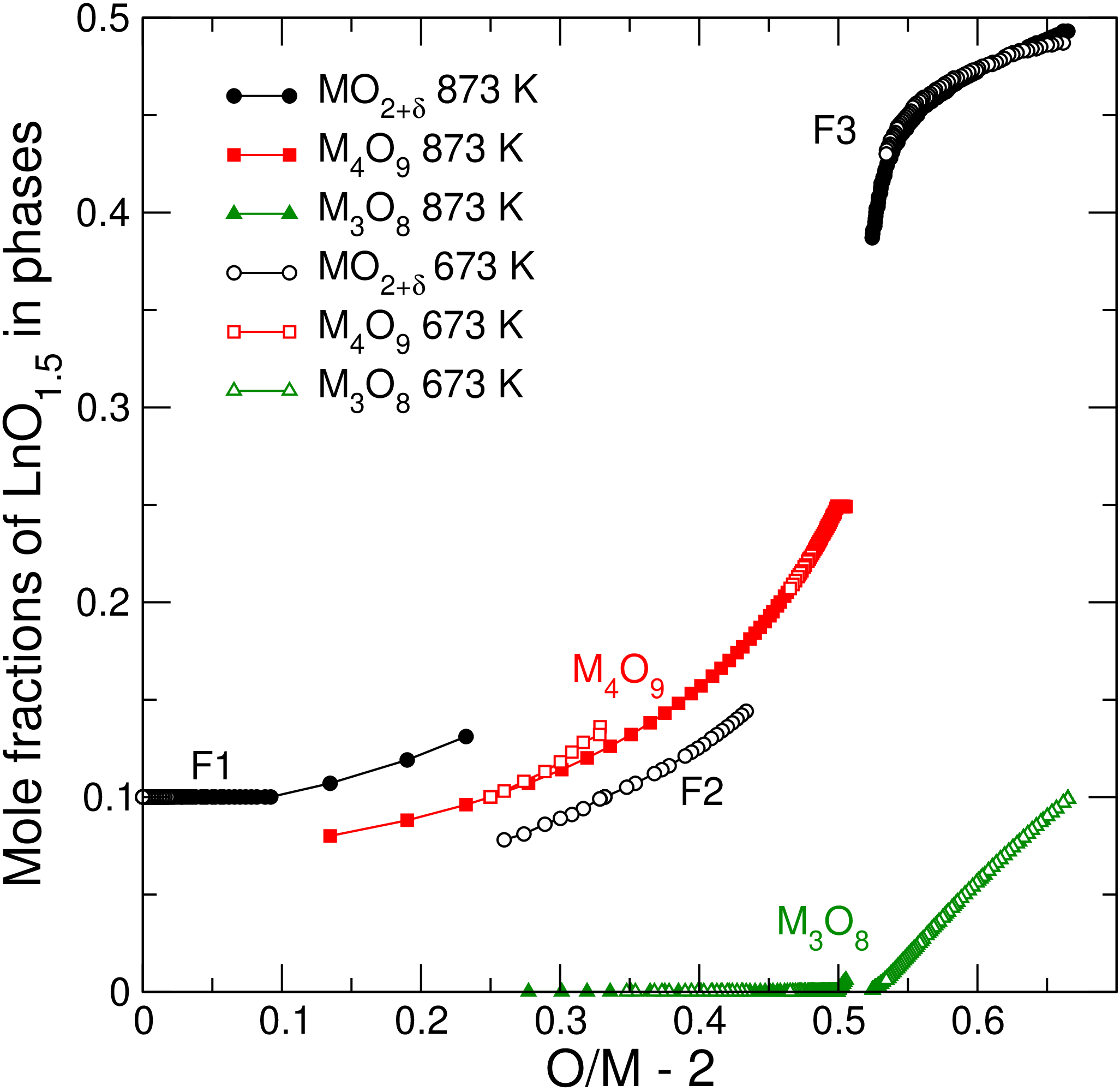


Fig. S4. Predicted evolution of composition of phases in a sample containing 10 mole % of *Ln*O_1.5_ in the process of equilibrium oxidation at 673-873 K. Filled and empty symbols correspond to 873 K and 673 K isotherms, respectively.

The developed thermodynamic model being combined with the ion-packing model allows predicting the variation of the lattice parameter as a function of *T*, *z*, and $\log\left( {P_{O_{2}}}/{P^{0}} \right)$ in any *Ln*O_1.5_-UO_2_-UO_3_ system, provided the set of ionic radii of *Ln*^+3^ in 6-, 7-, and 8-fold coordination is known. Figure S5 is constructed with the set of the radii for La^+3^ from Table S2 and with the thermodynamic parameters from Table S1.


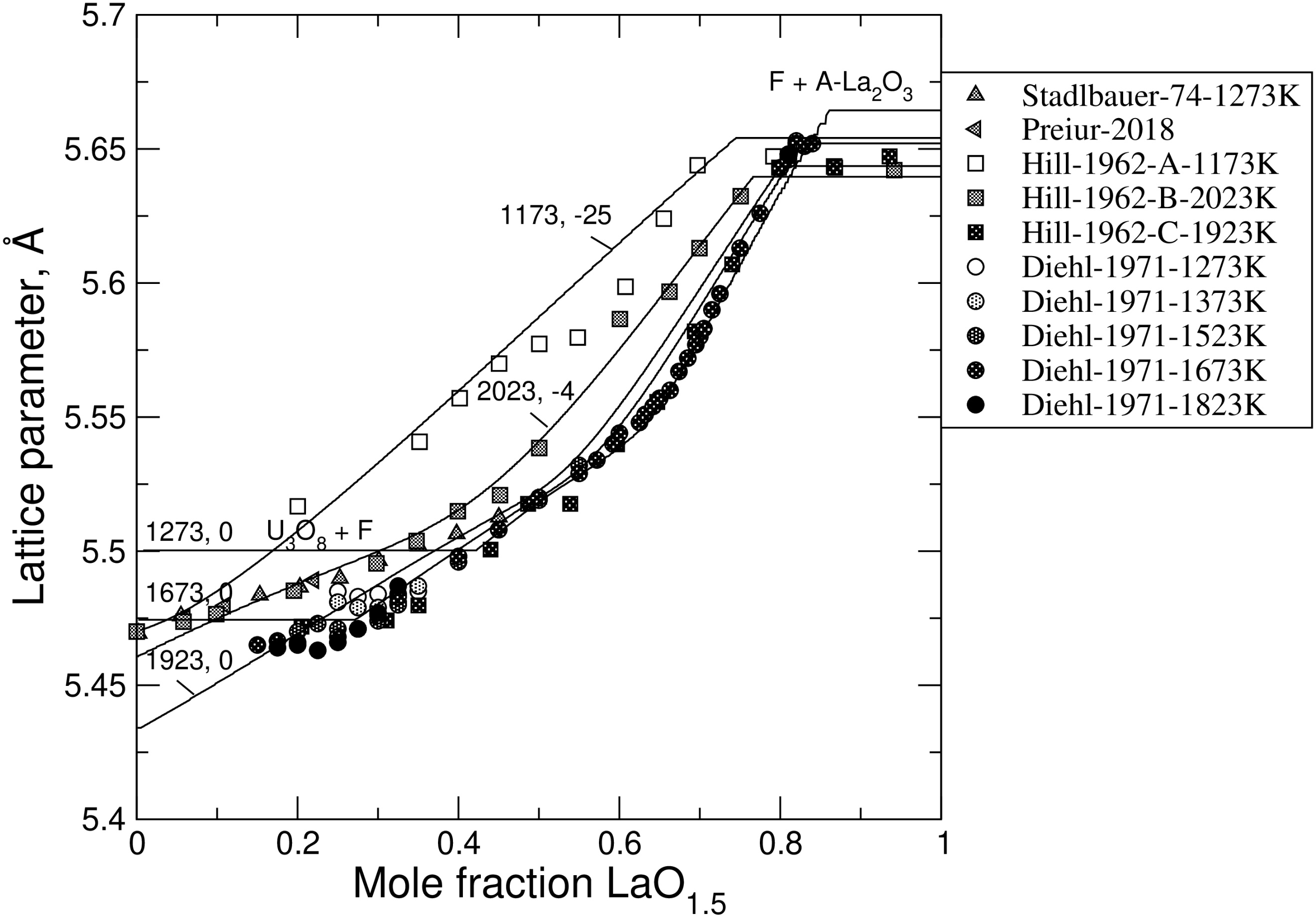


Fig. S5. Variation of the lattice parameter in UO_2_-LaO_1.5_ solid solutions predicted from the thermodynamic model combined with an ion-packing model. Solid lines correspond to equilibrium *a* vs. *z* relationships computed at fixed values of the temperature and the partial pressure of oxygen. Different lines emulate conditions of synthesis that correspond to experimental studies. The experimental data are from Stadlbauer et al. [7], Preiur et al. [8], Hill [9] and Diehl & Keller [10].

Figure S6 shows the evolution of endmember fractions in the fluorite-structured phase at 1273 K with the increase in the oxidation state. As $\log\left( {P_{O_{2}}}/{P^{0}} \right)$ increases from −25 to 0, the fractions of UO_2.5_, ${U_{1/2}{Ln}_{1/2}O}_{2}$and ${U_{1/3}{Ln}_{2/3}O}_{2}$endmembers increase. The UO_2.5_ endmember is important within the range of 0 < *z* < 0.5, while the ${Ln}_{2/3}{U_{1/3}O}_{2}$endmember is important within the range of 0.5 < *z* < 1. Vacancies could be completely avoided in strongly oxidized samples only within the interval of 0 < *z* < 0.67. The increase in the lattice parameter at *z* > 0.67 observed in UO_2_-LaO_1.5_ system in oxidized samples (Fig. S5) is likely due to the admixture of vacancies. The effective radius of the oxygen vacancy is larger than the radius of the oxygen anion by about 12 %, consistently with the steeper slope.


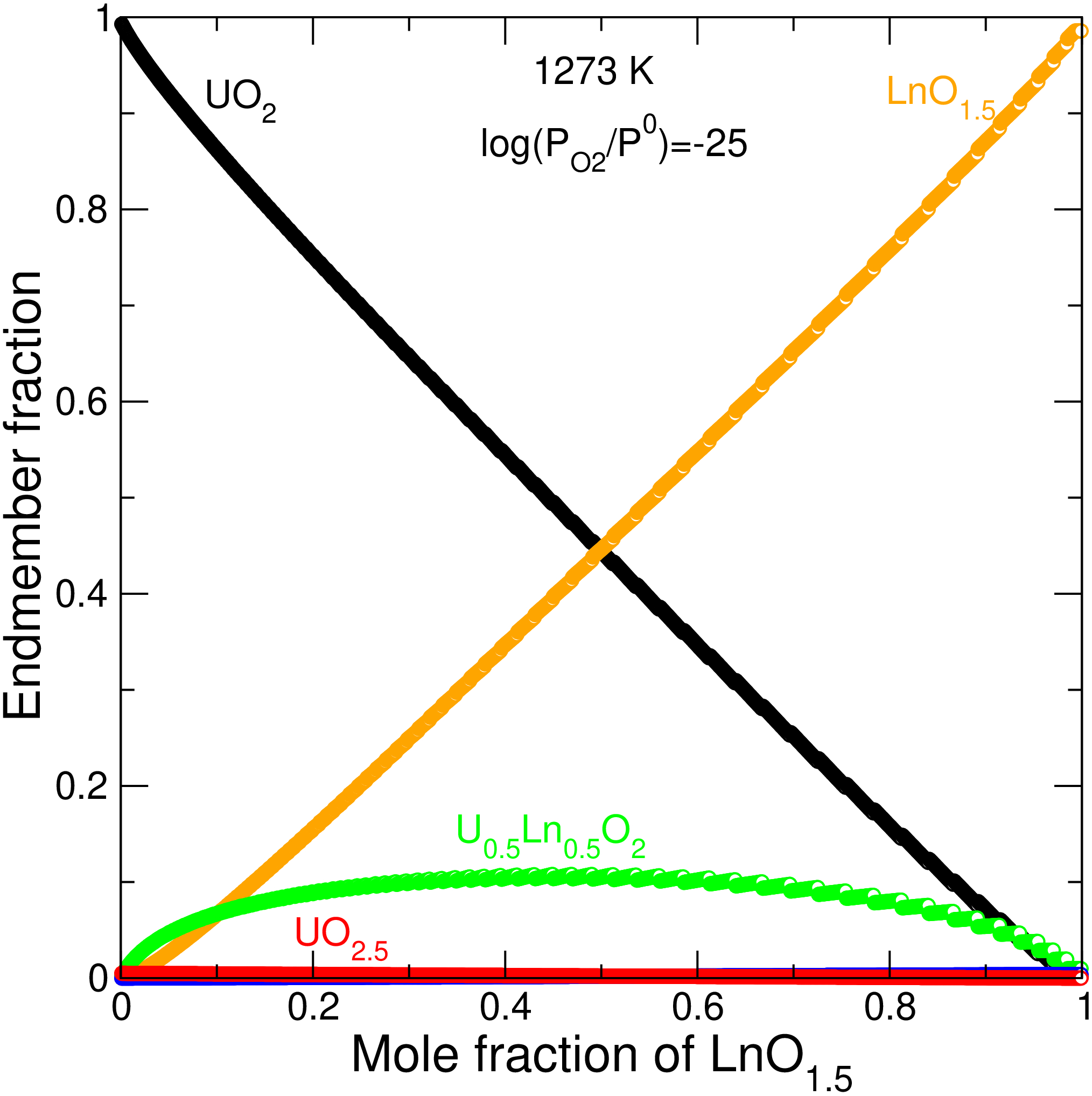

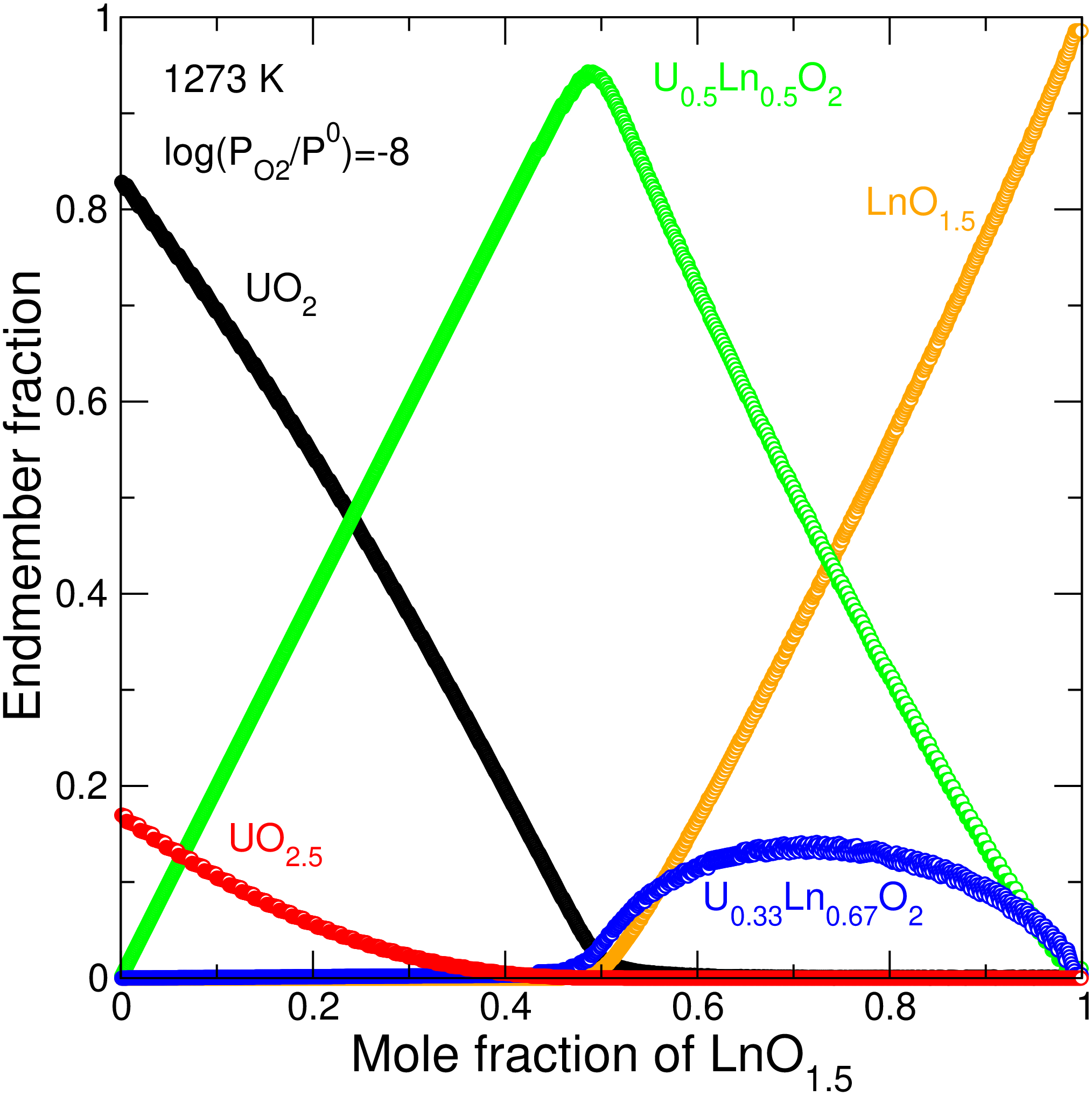

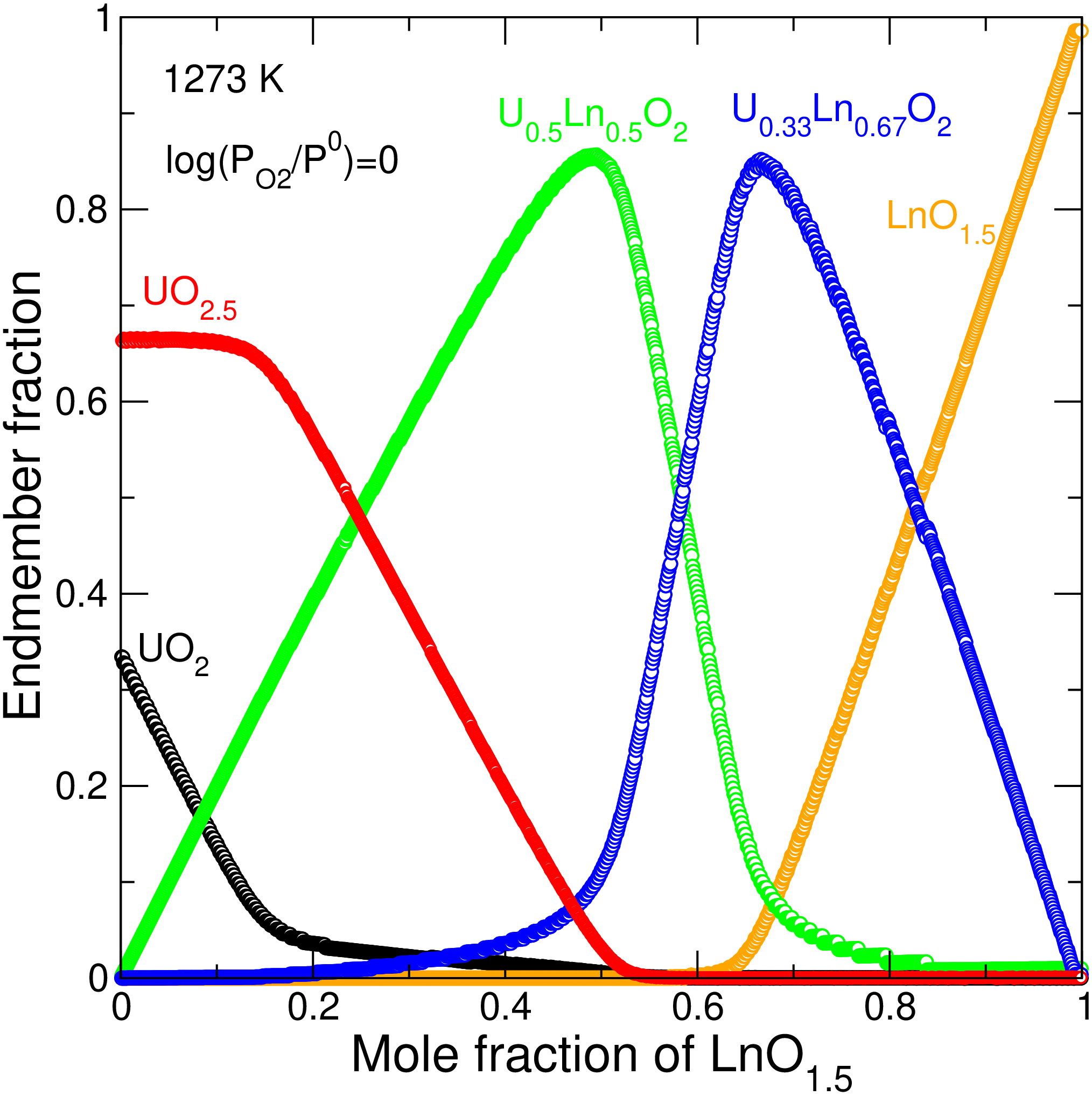


Fig. S6. Composition of the endmember fractions in fluorite at strongly reduced ($\log\left( {P_{O_{2}}}/{P^{0}} \right)=-25$), slightly oxidized ($\log\left( {P_{O_{2}}}/{P^{0}} \right)=-8$) and strongly oxidized ($\log\left( {P_{O_{2}}}/{P^{0}} \right)=0$) conditions. All other phases are suppressed.

Figure S7 illustrates a stepwise scheme of construction of the parameter space of MO_2+_*_δ_* fluorite phase which allows a consistent definition of endmember fractions. The endmembers UO_2.5_, ${U_{1/2}{Ln}_{1/2}O}_{2}$and ${U_{1/3}{Ln}_{2/3}O}_{2}$ are built via reactions between UO_2_ and *Ln*O_1.5_ components. The fractions of these endmembers are defined with the aid of the reaction progress variables *x*, *r* and *d*. The initial fractions of UO_2_ and *Ln*O_1.5_ are set 1 – *z* and *z*, respectively. The formation of ${Ln}_{1/2}{U_{1/2}O}_{2}$ consumes *r* moles of UO_2_ and *r* moles of *Ln*O_1.5_, while the formation of ${Ln}_{2/3}{U_{1/3}O}_{2}$ consumes *d* moles of UO_2_ and *2d* moles of *Ln*O_1.5_. A certain fraction of UO_2_ that is left free after the formation of ${U_{1/2}{Ln}_{1/2}O}_{2}$and ${U_{1/3}{Ln}_{2/3}O}_{2}$, may then transform into UO_2.5_. The fractions of the endmembers after the four steps become: *X*(UO_2_) = 1 – *z – r* – *d* – *x*, *X*(*Ln*O_1.5_) = *z – r* – 2*d*, *X*(${U_{1/2}{Ln}_{1/2}O}_{2}$) = 2*r*, *X*(${U_{1/3}{Ln}_{2/3}O}_{2}$) = 3*d*, *X*(UO_2.5_) = *x* . The equilibrium values of *x*, *r* and *d* are determined via minimization of the Gibbs free energy of the fluorite phase with respect to these variables.


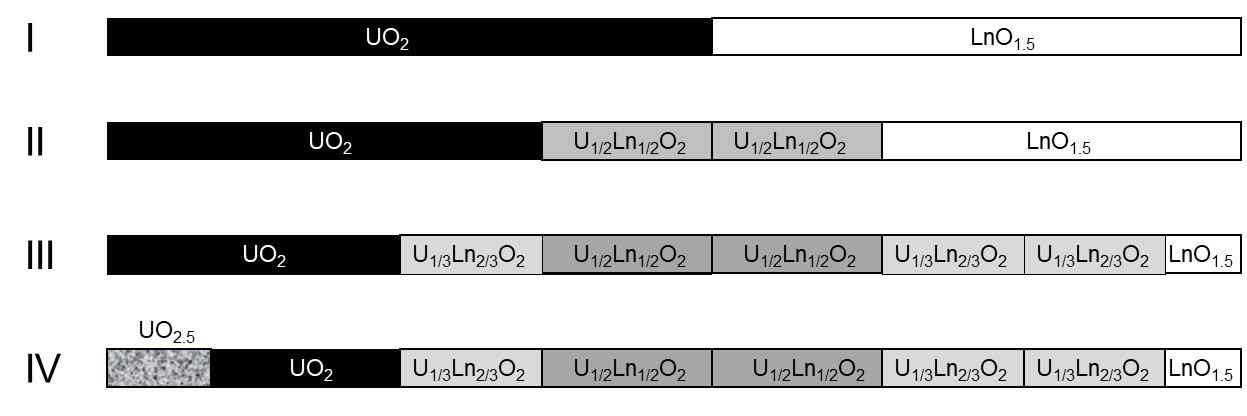


Fig. S7. Scheme of construction of the parameter space of the fluorite phase.

Table S2. Cation radii (in Å) accepted in this study.

| $C^{Q}$ | $R_{C}^{Q,6}$ | $R_{C}^{Q,7}$ | $R_{C}^{Q,8}$ | $R_{C}^{Q,9}$ |
| --- | --- | --- | --- | --- |
| Y^+3^ | 0.8820 | 0.9650 | 1.0170 |  |
| Gd^+3^ | 0.9380 | 1.0012 | 1.0560 |  |
| Nd^+3^ | 0.9880 | 1.0530 | 1.1030 |  |
| La^+3^ | 1.0600 | 1.1300 | 1.1670 |  |
| U^+4^ | 0.8900 | 0.9430 | 0.9952 |  |
| U^+5^ | 0.7600 | 0.8130 | 0.8653 | 0.9630 |
| U^+6^ | 0.6500 | 0.7023 | 0.7550 |  |
| Pu^+3^ | 1.0 | 1.0658 | 1.1136 |  |
| Pu^+4^ | 0.86 | 0.9115 | 0.96294 |  |

Note: These cation radii should be used together with the values of $R_{O}=1.3736$ Å and $R_{V}=1.5410$ Å, which refer to the lattice oxygen anion and the oxygen vacancy, respectively.

1. Chartier, A., Van Brutzel, L., Fossati, P., Martin, Ph., and Guéneau, Chr. Thermodynamic and Thermophysical Properties of the Actinide Oxides. In Comprechensive Nuclear Materials (Second Edition) Volume 7. Eds: Konings, R. J. M., Stoller, R.E. and Agarwal, R., Pp. 111-154, 2020, Elsevier. <https://doi.org/10.1016/B978-0-12-803581-8.11786-2>

2. Lindemer, T. B. & Sutton, Jr. A. L. Study of nonstoichiometry of U_1–z_Gd_z_O_2±x_. *J. Am. Ceram. Soc*. **71**, 553-561 (1988). doi: <https://doi.org/10.1111/j.1151-2916.1988.tb05919.x>

3. Yoshida K., Arima T., Inagaki Y., Idemitsu K., Osaka M., Miwa S. Oxygen potential of hypo-stoichiometric La-doped UO_2_. *J. Nucl. Mater.***418**, 22-26 (2011). <https://doi.org/10.1016/j.jnucmat.2011.06.045>

4. Hagemark K., Broli M. Equilibrium oxygen pressures over solid solutions of urania-yttria and urania-lanthana at 1100° to 1400°C. *J. Am. Ceram. Soc.* **50**, 563-567 (1967). <https://doi.org/10.1111/j.1151-2916.1967.tb14999.x>

5. Une K., Oguma M. Oxygen potentials of (U,Nd)O_2 ± x_ solid solutions in the temperature range 1000–1500°C. *J. Nucl. Mater.* **118**, 189-194 (1983). <https://doi.org/10.1016/0022-3115(83)90224-6>

6. Kim J.-G., Ha Y.-K., Park S.-D., Jee K.-Y., Kim W.-H. Effect of a trivalent dopant, Gd^3+^, on the oxidation of uranium dioxide. *J. Nucl. Mater.* **297**, 327-331 (2001). <https://doi.org/10.1016/S0022-3115(01)00639-0>

7. Stadlbauer, E., Wichmann, U., Lott, U. & Keller, C. Thermodynamics and phase relationships of the ternary lanthanum-uranium-oxygen system. *J. Solid State Chem*. **10**, 341-350 (1974). <https://doi.org/10.1016/0022-4596(74)90043-7>

8. Prieur D., Martel L., Vigier J.-F., Scheinost A. C., Kvashnina K. O., Somers J., et al. Aliovalent cation substitution in UO_2_: Electronic and local structures of U_1–y_La_y_O_2±x_ solid solutions. *Inorg Chem.* **57,** 1535-44 (2018). <https://doi.org/10.1021/acs.inorgchem.7b02839>

9. Hill D. C. Phase relations and crystal chemistry in the system uranium oxide–lanthanum oxide. *J. Am. Ceram. Soc*. **45**, 258-263 (1962). <https://doi.org/10.1111/j.1151-2916.1962.tb11140.x>

10. Diehl, H.G. & Keller, C. Das System UO_2_-UO_3_-LaO_1.5_. *J. Solid State Chem*., **3**, 621-636 (1971). <https://doi.org/10.1016/0022-4596(71)90110-1>
